# Transcriptional Profile of *Aedes aegypti* Leucine-Rich Repeat Proteins in Response to Zika and Chikungunya Viruses

**DOI:** 10.3390/ijms20030615

**Published:** 2019-01-31

**Authors:** Liming Zhao, Barry W. Alto, Dongyoung Shin

**Affiliations:** Florida Medical Entomology Laboratory, University of Florida, 200 9th Street South East, Vero Beach, FL 32962, USA; bwalto@ufl.edu (B.W.A.); dshin@ufl.edu (D.S.)

**Keywords:** *Aedes aegypti*, Leucine-Rich Repeat Proteins, Zika virus, chikungunya virus, immune responses, gene expression

## Abstract

*Aedes aegypti* (L.) is the primary vector of chikungunya, dengue, yellow fever, and Zika viruses. The leucine-rich repeats (LRR)-containing domain is evolutionarily conserved in many proteins associated with innate immunity in invertebrates and vertebrates, as well as plants. We focused on the *AaeLRIM1* and *AaeAPL1* gene expressions in response to Zika virus (ZIKV) and chikungunya virus (CHIKV) infection using a time course study, as well as the developmental expressions in the eggs, larvae, pupae, and adults. RNA-seq analysis data provided 60 leucine-rich repeat related transcriptions in *Ae. aegypti* in response to Zika virus (Accession number: GSE118858, accessed on: August 22, 2018, GEO DataSets). RNA-seq analysis data showed that *AaeLRIM1* (AAEL012086-RA) and *AaeAPL1* (AAEL009520-RA) were significantly upregulated 2.5 and 3-fold during infection by ZIKV 7-days post infection (dpi) of an *Ae. aegypti* Key West strain compared to an Orlando strain. The qPCR data showed that LRR-containing proteins related genes, *AaeLRIM1* and *AaeAPL1*, and five paralogues were expressed 100-fold lower than other nuclear genes, such as defensin, during all developmental stages examined. Together, these data provide insights into the transcription profiles of LRR proteins of *Ae. aegypti* during its development and in response to infection with emergent arboviruses.

## 1. Introduction

The leucine-rich repeats (LRR)-containing domain is noted to be evolutionarily conserved among many proteins correlated with innate immunity in an array of organisms, including invertebrates, vertebrates, and plants [1]. Innate immunity is a conserved host response that serves as the host’s first line of defense by sensing pathogen-associated molecular patterns through germline-encoded pattern recognition receptors [2]. For example, in mammals, activation of antimicrobial defenses and stimulation of the adaptive immune response is mediated by Toll-like receptors (TLRs) and nucleotide-binding oligomerization domain (NOD)-like receptors through the LRR domain in response to pathogens [3].

Mosquitoes lack an adaptive immune system [4] and so they are fully dependent on mounting an innate immune response to fight infection [5,6,7,8]. Research indicates that mosquito vectors and mosquito cell lines produce both humoral and cellular immune responses against invading pathogens [9,10]. Leucine-rich repeat immune proteins (LRIMs) are a mosquito-specific family of putative innate receptors [11,12,13]. Two LRR proteins, LRIM1 (leucine-rich repeat immune protein 1) and APL1 (Anopheles Plasmodium-responsive leucine-rich repeat 1), have been recognized as major mosquito factors that regulate parasite infection and parasite loads [14]. In *Anopheles* mosquitoes, *LRIM1*, *APL1C*, and LRR-containing proteins activate complement-like defense responses against malaria by forming a disulphide-bridge that interacts with thioester-containing protein 1 (TEP1), a complement C3-like protein [15,16]. *APL1* of *Anopheles gambiae* is a family of variable LRR proteins required for cell-mediated protection as shown using a rodent model malaria parasite, *Plasmodium berghei* [17]. The *An. gambiae APL1* genomic locus circumscribes three distinct genes (*APL1A*, *APL1B*, and *APL1C*), however, only the product of *APL1C* acts as a *P. berghei* antagonist [17]. *LRIM1* and *APL1C* both play a significant role in the anti-*Plasmodium* response. Accordingly, silencing of these genes results in an altered response against *Plasmodium* infection [13]. A genome-wide study found that variations or polymorphisms in the *LRIM1* and *APL1C* proteins were correlated with resistance and susceptibility to *Plasmodium* infection [18]. Additional studies offered mechanisms for controlling *Plasmodium* early during the infection process by targeting the ookinete or oocyst stages of oocyst [19,20]. Nonetheless, no detailed information has been generated for pathogen response by the LRR-containing proteins of immune factors in *Aedes* mosquitoes, responsible for transmitting multiple emerging arboviruses.

*Aedes aegypti* (L.) is a vector for transmitting emergent arboviruses, including chikungunya, dengue, yellow fever, and Zika viruses. Chikungunya fever is primarily transmitted to humans through mosquito vectors, *Ae. aegypti* and *Ae. albopictus*. It is a viral disease belonging to the family, Togaviridae, and genus, *Alphavirus*. Recent outbreaks of chikungunya fever occurred from 2004 to 2006 spanning Kenya in 2004 (Eastern/Central/Southern African, ECSA, CHIKV lineage) and the island of La Réunion in 2005-2006 (Indian Ocean CHIKV lineage). It later emerged in the New World in 2013 on St. Martin Island (Asian CHIKV lineage), eventually spreading throughout the Americas [21,22,23,24]. Over the span of the past 12 years, it is estimated that more than four million human cases of chikungunya infection have occurred worldwide [25]. Symptoms of infection include a rash, fever, headache, joint pain, and muscle pain [26] along with the chance of developing chronic musculoskeletal diseases [27]. There is currently no vaccine available for CHIKV. Accordingly, controlling the mosquito vectors is the primary method utilized to reduce the risk of disease transmission.

Zika virus (ZIKV) was first discovered in 1947 and belongs to the family, Flaviviridae, genus, *Flavivirus* (CDC 2016) [28]. Spreading to the Oceania region, ZIKV caused outbreaks on Yap Island in Micronesia and French Polynesia in 2007 and 2013, respectively. In 2015, ZIKV was found to have reached Brazil, spreading throughout the Americas [28,29]. It is estimated that 1.5 million people have been infected by ZIKV in Brazil [30]. ZIKV continues to spread to new areas. Transmission in the U.S. is a major public health risk, notably for the Gulf states, such as Florida and Texas, where ecological conditions are favorable for the primary vector, *Ae. aegypti*, as well as an increasing likelihood for virus introduction by imported cases. Manifestations of ZIKV take on different forms, the most serious of which include birth defects in humans [31] along with neurological complications that may result in Guillain-Barré syndrome, both of which are significant public health threats [32].

Mosquitoes respond to infection using an array of molecular signaling pathways and immune effector proteins. A focus on the immune system response of *Ae. aegypti* has unveiled a transcriptome analysis of genome-wide mechanisms that are implicated in defense against arbovirus infections [33,34,35,36,37]. No sequence-structure-function relationships of mosquito leucine-rich repeat immune proteins in *Ae. aegypti* in response to arboviruses are available, though the LRR-proteins have been compared with *An. gambiae* and *Culex quinquefasciatus* [16]. Many studies of *LRIM1* and *APL1C* have been reported as playing an important role in defense against *Plasmodium* in *Anopheles* [13,14,15]. However, there is no study of *LRIM1* and *APL1C* in the *Aedes* in response to arboviruses. Therefore, we re-examined previous RNAseq data analysis and obtained 60 leucine-rich repeat related transcriptions of *Ae. aegypti* (Accession number: GSE118858, accessed on: August 22, 2018, https://www.ncbi.nlm.nih.gov/gds/?term=GSE118858) [34]. We examined the *AaeLRIM1* and *AaeAPL1* gene expressions in response to both ZIKV and CHIKV infection using a time course study. Additionally, we investigated the developmental expressions of these genes in the eggs, larvae, pupae, and adults. The current study aims to improve our understanding of the transcription profiles of *Ae. aegypti* LRR proteins during development and in response to arbovirus infection.

## 2. Results

### 2.1. Leucine-Rich Repeat Proteins Changes in Transcriptome of the Ae. aegypti Female Adult in Response to ZIKV Infection

To better our understanding of molecular interactions and the immune response to arbovirus of *Ae. aegypti* from Florida, we re-examined RNA-seq data to explore the changes in leucine-rich repeat proteins in the *Ae. aegypti* (Key West and Orlando strains) transcriptome in response to oral ingestion of ZIKV infected blood and ZIKV infection (Accession number: GSE118858, https://www.ncbi.nlm.nih.gov/gds/?term=GSE11). Data were provided by RNA-seq analysis that generated 60 leucine-rich repeat related transcriptions in the *Ae. aegypti* genome in response to Zika virus (Table S1A–D and Table 1A–D). Specifically, female *Ae. aegypti* transcriptomic RNA-seq data showed that 23 genes related to leucine-rich repeat proteins (LRRP) were significantly upregulated during infection by ZIKA in 7-days post infection (dpi) *Ae. aegypti* Key West strains compared with Orlando strains. Additionally, 17 of these genes between the two strains were upregulated more than 2-fold (p-adj ≤ 0.01; log2 fold change > ±2.0) in response to ZIKV 7 dpi (Table 1A). *AaeLRIM1* (AAEL012086-RA) and *AaeAPL1* (AAEL009520-RA) were significantly upregulated 2.5 and 3-fold (Table 1A). When comparing transcriptome profiles of two *Ae. aegypti* strains in response to the control (blood-feeding only), only three genes related to leucine-rich repeat proteins were significantly upregulated/downregulated in 7-days post infection *Ae. aegypti* in Key West strains compared with Orlando strains (Table 1B). *AaeLRIM1* (AAEL010286-RA) was significantly upregulated 3-fold (Table 1B). Comparing ZIKV infected Key West *Ae. aegypti* with the Key West control at 7 dpi, four differentially expressed (DE) transcripts related to LRRP were significantly dysregulated (two upregulated and two downregulated, Table 1C). *AaeLRIM1* (AAEL010286-RA, p-adj 5.0 × 10^−9^, log2 fold change −3.3918) was significantly down-regulated (Table 1C). Analysis and comparison of mRNA expression profiles of *Ae. aegypti* Orlando strains following ZIKV infection indicated five LRRP related genes, including *AaeAPL1* (AAEL009520-RA, p-adj 2.8 × 10^−4^, log2 fold change −2.2074), were significantly dysregulated (downregulated) 7-days post infection (Table 1D).

### 2.2. Leucine-Rich Repeat Proteins AaeAPL1 Paralogues of Aedes aegypti

Evolutionary analysis of eight paralogues of *AeaAPL1* of the *Ae. Aegypti* were conducted in MEGA7 [38] (Figure S1). The data showed that *AaeAPL1* was closely related to *AaeLRIM4*. The DNA sequences producing significant alignments between *AaeAPL1* and *AaeLRIM4* showed 44% identity.

### 2.3. Developmental Regulation of AaeLRIM1 and AaeAPL1

**Figure 1:**
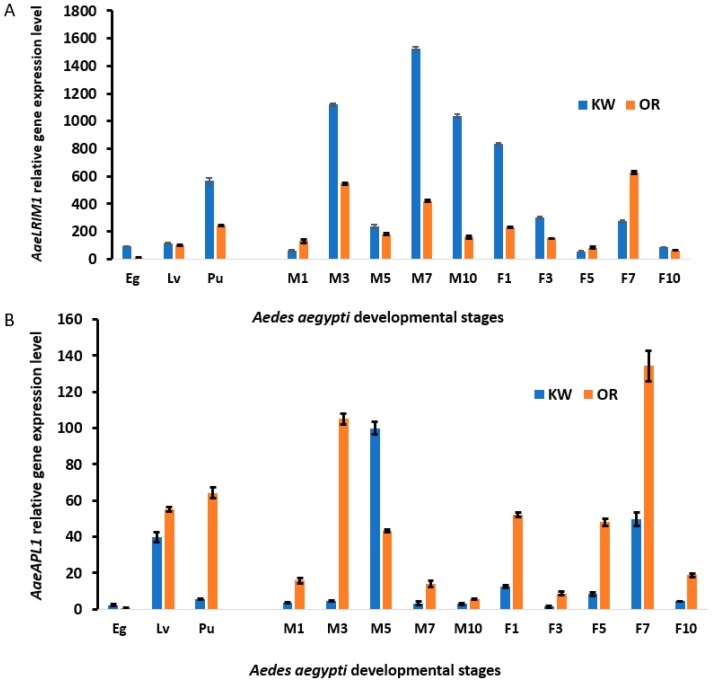
*AaeLRIM1* and *AaeAPL1* relative expression in the all developmental stages, from eggs (Eg), larvae (Lv), pupae (Pu), and adults *Ae. aegypti*, including male (M1, male 1-d-old; M3, male 3-d-old; M5, male 5-d-old; M7, male 7-d-old; and M10, male 10-d-old) and female (F1, female 1-d-old; F3, female 3-d-old; F5, female 5-d-old; F7, female 7-d-old; and F10, female 10-d-old) in the Key West strain (KW) and Orlando (OR) strain of *Aedes aegypti*. (**A**) *AaeLRIM1*; (**B**) *AaeAPL1*.

To understand how *AaeLRIM1* and *AaeAPL1* are regulated during the development of *Ae. aegypti*, qPCR was performed to examine the relative transcription levels of *AaeLRIM1* and *AaeAPL1* in eggs, larvae, pupae, and male and female adults (Figure 1A,B). 

In addition, we also examined five paralogues of *AeaAPL1*, i.e., *AaeLRIM3, AaeLRIM4*, *AaeLRIM15*, *AaeLRIM16*, and *AaeLRIM17*, during developmental stages using qPCR (Figure S2A–E).

#### 2.3.1. *AaeLRIM1* and *AaeAPL1* RNA Profile in Immature Stages of *Ae. aegypti*

**Figure 2:**
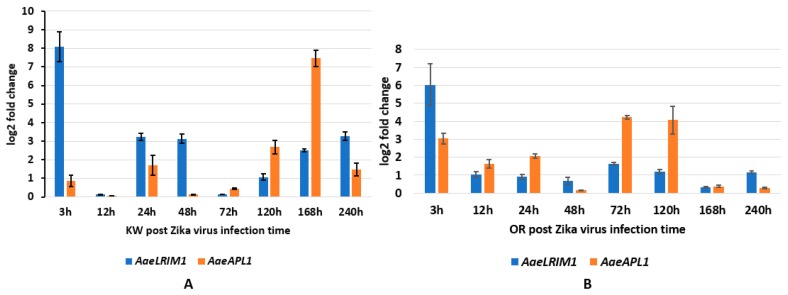
*AaeLRIM1* and *AaeAPL1* relative expression level fold changes in *Aedes aegypti* female infected with ZIKV. The fold change was calculated using the 2 ^[−average *ΔΔ*CT]^ method. *Δ*Ct (Control) = Ct (*AaeLRIM1*/*AaeAPL1*) − Ct (*AeaActin*); *Δ*Ct (infected-ZIKV) = Ct (*AaeLRIM1*/*AaeAPL1*) − Ct (*AeaActin*); *ΔΔ*Ct =*Δ*Ct (infected-ZIKV) − *Δ*Ct (Control). The 3, 12, 24, 48, 72, 120, 168, and 240 h represented gene expression post infection with ZIKV. (**A**) Key West strain female *Ae. aegypti*; (**B**) Orlando strain female *Ae. aegypti*.

Multivariate analysis of variance (MANOVA) showed significant effects of mosquito strain, developmental stage, and interaction of these factors (Table 2A). For the mosquito strain effect, Key West (permethrin resistant) had higher *AaeLRIM1* than Orlando (permethrin susceptible strain) *Ae. aegypti* [34] (Figure 2). Standardized canonical coefficients showed that *AaeLRIM1* and *AaeAPL1* contributed similarly, but in opposite directions (Table 2A). In contrast, Orlando had higher *AaeAPL1* than Key West *Ae. aegypti* (Figure 1). Standardized canonical coefficients showed that *AaeLRIM1* contributed twice as much as AaeAPL1 for the significant developmental stage effect (Figure 2). For the developmental stage effect, *LRIM1* was significantly different between developmental stages with increases associated between each stage (Figure 1). Gene expression of *AaeAPL1* was significantly higher for the larval and pupal stages compared to the egg stage. However, *AaeAPL1* was lower among pupae than larvae (Figure 1). For the significant interaction, we compared less than all possible treatment groups by which developmental stage was compared within a given strain (e.g., Key West eggs vs. Key West larvae). Standardized canonical coefficients showed a similar contribution of *AaeLRIM1* and *AaeAPL1* to the significant interaction, in opposite directions (Table 2A). Gene expression of *AaeLRIM1* was higher for Key West than Orlando *Ae. aegypti* and occurred over a greater range. We observed significant increases in gene expression of *AaeLRIM1* for each developmental stage for both Key West and Orlando *Ae. aegypti* (Figure 2). In contrast, gene expression of *AaeAPL1* was higher for Orlando than Key West *Ae. aegypti* and occurred over a greater range. Gene expression of *AaeAPL1* significantly increased for each developmental stage for both Key West and Orlando *Ae. aegypti* (Figure 2).

#### 2.3.2. *AaeLRIM1* and *AaeAPL1* RNA Profile in Adults (Male and Female) of *Ae. aegypti*

MANOVA showed significant main effects of mosquito strain, sex, and age, as well as the two-way interaction and three-way interaction (strain **×** sex **×** age). Standardized canonical coefficients showed that *AaeLRIM1* contributed much more to all significant effects than *AaeAPL1* (Table 2B). Because the three-way interaction was significant, we focused on pairwise comparisons of treatment groups for this effect. Specifically, we compared mosquito strains of a given sex and age (e.g., Key West, female, 1-day old vs. Orlando, female, 1-day old). For expression of *AaeLRIM1*, Key West was higher for 1-day, 3-day, and 10-day old mosquitoes than Orlando female *Ae. aegypti*. In contrast, *AaeLRIM1* was higher for 5-day and 7-day Orlando than Key West female *Ae. aegypti*. Gene expression for *AaeLRIM1* was higher for all ages of Key West male *Ae. aegypti* except for 1-day old males. Lowest rates of expression were observed for 1-day old male *Ae. aegypti* for both strains and highest rates were observed for intermediate aged mosquitoes.

For gene expression of *AaeAPL1*, Orlando was higher for all ages than Key West female *Ae. aegypti*. Rates of expression were highest for 7-day old female mosquitoes and expression was lower among older females (10-day old) for both mosquito strains. For gene expression of *AaeAPL1*, Orlando was higher for all ages than Key West male *Ae. aegypti*, except 5-day old males. Rates of expression were highest for 5-day old and 3-day old male mosquitoes for Key West and Orlando *Ae. aegypti*, respectively. Gene expression was low for young and old male mosquitoes (Figure 1).

### 2.4. Infection in Ae. aegypti Exposed to CHIKV and ZIKV

In Florida, and other geographic regions of the world, there is a heterogeneity in the susceptibility of *Ae. aegypti* to insecticides, in part, attributable to the evolution of insecticide resistance. In particular, pyrethroid insecticides, such as permethrin, continue to be the primary means to control *Ae. aegypti* [39]. For this study, we deliberately included both susceptible and permethrin resistant strains of *Ae. aegypti* to allow for the possibility to observe differences in gene expression between the strains following ingestion of arbovirus infected blood. The susceptible strain was a long-standing laboratory colony of *Ae. aegypti* originating from Orlando, Florida (28.53° N, 81.37° W). The resistant strain was created based on a collection of *Ae. aegypti* from Key West, Florida (24.55° N, 81.78° W). We first tested *Ae. aegypti* from Key West for permethrin resistance following the use of World Health Organization (WHO) bottle bioassays (WHO 2016). Next, we subjected this population to 15 generations of permethrin selection and re-tested for permethrin resistance (resistant strain). *Aedes aegypti* females aged 4-days as adults were allowed to feed on blood consisting of either 6.4 log10 pfu/mL of ZIKV (Table 3A) or 8.0±0.09 and 8.3 ± 0.08 log10 pfu/mL of CHIKV for each strain (Table 3B). The amount of virus imbibed by mosquitoes and the viral titer at 3, 7, and 10, days post infections are described in Table 3A, B. After 10-days post infection, but not at any earlier times, we detected 100-fold higher ZIKV titer in the permethrin resistant strain than the susceptible strain (t4 = 8.12, *p* = 0.001) [34]. For the CHIKV infection study, we observed no significant differences in the viral titer between the susceptible and resistant strains of *Ae. aegypti* (all *p* > 0.05). 

### 2.5. AaeLRIM1 and AaeAPL1 Transcriptional Induction of ZIKV Infections in Orally Infected Ae. aegypti Females

To characterize *AaeLRIM1* and *AeaAPL1* expression in response to ZIKV exposure, we measured *AaeLRIM1* and *AeaAPL1* expressions in orally infected *Ae aegypti*. MANOVA showed the significant effects of strain of *Ae. aegypti*, time, and their interaction (Table 4A). For the strain effect, SCCs showed that *AaeLRIM1* contributed more to the significant effect than *AaeAPL1* (Table 4A). Gene expression of *AaeLRIM1* was significantly higher for Key West than Orlando strains. Similar contributions of gene expression of *AaeAPL1* were observed for Orlando and Key West *Ae. aegypti*. For the significant time effect, SCCs showed that *AaeLRIM1* contributed approximately 8-fold higher than *AaeAPL1* (Table 4A). The highest gene expression of *AaeLRIM1* was 3-hours post infection with the later time points having lower levels. For *AaeAPL1*, the highest levels were observed for 72, 120, and 168-hours post infection with other points being lower (Figure 2).

For the interaction, SCCs showed that *AaeAPL1* contributed approximately 2-fold greater than *AaeLRIM1* (Table 4A). We made less than all possible comparisons by comparing between strains and holding time constant (e.g., Key West, 3-hours vs. Orlando, 3-hours). Gene expression for *AaeLRIM1* was highest for 3-hours post infection compared to other time points for both Key West and Orlando *Ae. aegypti*. All pairwise contrasts of treatment groups were significantly different from one another, except for 120 h post infection (Figure 2A,B).

For the significant interaction, gene expression for *AaeAPL1* was highest for Key West at 168-hours post infection and for Orlando at 72- and 120-hours post infection for *Ae. aegypti*. All pairwise comparisons were significantly different between each other except for 24- and 48-hours post infection between the two mosquito strains (Figure 2A,B). For comparison, the amount of ZIKV imbibed by mosquitoes (3-hours) and the viral titer at 72-hours, 168-hours, and 240-hours days post infection are described in Table 3A.

**Figure 3:**
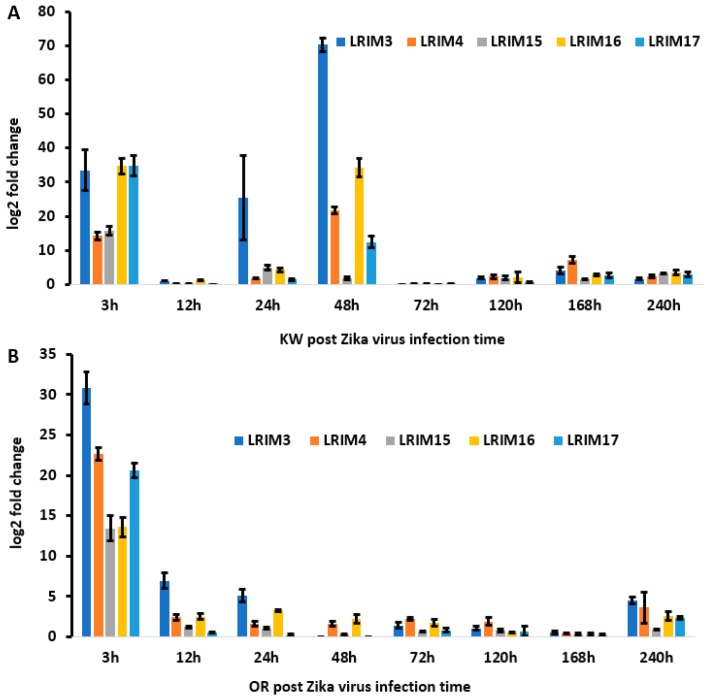
*AaeLRIM3, AaeLRIM4, AaeLRIM15, AaeLRIM16*, and *AaeLRIM17* relative expression level fold changes in *Aedes aegypti* female infected with ZIKV. The fold change was calculated using the 2 *^[−average ΔΔCT]^* method. *Δ*Ct (Control) = Ct (*AaeLRIM1*/*AaeAPL1*) – Ct (*AeaActin*); *Δ*Ct (infected-ZIKV) = Ct (*AaeLRIM1*/*AaeAPL1*) − Ct (*AeaActin*); *ΔΔ*Ct =*Δ*Ct (infected-ZIKV) − *Δ*Ct (Control). The 3, 12, 24, 48, 72, 120, 168, and 240 h represented gene expression post infected with ZIKV. (**A**) KW strain female *Ae. aegypti;* (**B**) Orlando strain female *Ae. aegypti*.

To determine paralogues of *AeaAPL1* expression in response to ZIKV exposure, we measured *AeaLRIM3*, *AeaLRIM4, AeaLRIM15, AeaLRIM16*, and *AeaLRIM17* expressions in *Ae. aegypti*. MANOVA showed significant effects of the strain of *Ae. aegypti*, time, and their interaction (Table 4B). For the strain effect, gene expression of all five paralogues of *AeaAPL1* were higher for Key West than Orlando strains, with *AeaLRIM16* being the largest contributor. For the time effect, *AeaLRIM17* made the largest contribution with the highest expression at 3 h post infection. Gene expression for the remaining paralogues were highest between 3- and 48-hours post infections (Figure 3A,B). 

For the interaction, SCCs showed that *AeaLRIM16* contributed approximately 2- to 8-fold greater than the other paralogues. As previously described, we made comparisons between strains and held time constant (e.g., Key West, 3-hours vs. Orlando, 3-hours). Gene expression for *AeaLRIM16* was significantly higher at 3- and 48-hour post infection for Key West than Orlando strains of *Ae. aegypti*. No other comparisons were significantly different from one another after adjusting the experimentwise alpha for multiple comparisons.

### 2.6. AaeLRIM1 and AaeAPL1 Transcriptional Induction of CHIKV Infections in Orally Infected Ae. aegypti Females

**Figure 4:**
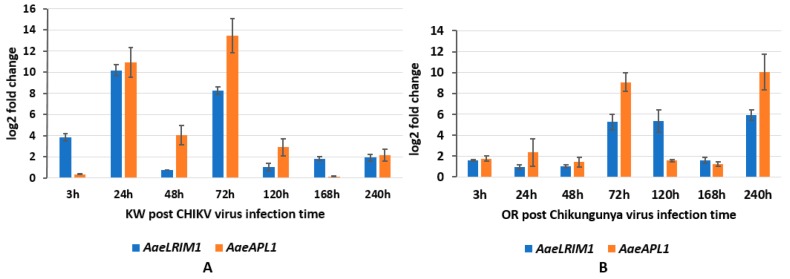
*AaeLRIM1* and *AaeAPL1* relative expression level fold changes in *Aedes aegypti* female infected with CHIKV. The fold change was calculated using the 2 *^[−average ΔΔCT]^* method. *Δ*Ct (Control) = Ct (*AaeLRIM1*/*AaeAPL1*) − Ct (*AeaActin*); *Δ*Ct (infected-CHIKV) = Ct (*AaeLRIM1*/*AaeAPL1*) − Ct (*AeaActin*); *ΔΔ*Ct =*Δ*Ct (infected-CHIKV) − *Δ*Ct (Control). The 3, 24, 48, 72, 120, 168, and 240 h represented gene expression post infected with CHIKV. (**A**) Key West strain female *Ae. aegypti*; (**B**) Orlando strain female *Ae. aegypti*.

To characterize *AaeLRIM1* and *AaeAPL1* expression in response to CHIKV exposure, we measured *AaeLRIM1* and *AaeAPL1* expressions in orally infected *Ae aegypti*. MANOVA showed the significant effects of the strain of *Ae. aegypti*, time, and their interaction. For all significant treatment effects, SCCs showed that *AaeLRIM1* contributed approximately 2-6-fold greater than *AaeAPL1* (Table 5A). For the strain effect, gene expression of *AaeLRIM1* and *AaeAPL1* was significantly higher for Key West than Orlando strains. For the time effect, gene expression of *AaeLRIM1* was highest at 24-hours post infection. All time points were significantly different from one another, except 3-hours versus 120-hours post infection (Figure 4). Similarly, gene expression of *AaeAPL1* was highest at 72-hours post infection. All time points were significantly different from one another except the following: 3-hours versus 168-hours, 24-hours versus 240-hours, and 48-hours versus 120-hours (Figure 4).

For the significant interaction, gene expression of *AaeLRIM1* was highest for 24-hours and 240-hours post infection for Key West and Orlando *Ae. aegypti*, respectively (Figure 4). All pairwise comparisons of treatment groups were significantly different from one another except for 48-hours and 168-hours post infection (Figure 4). The timing of the highest gene expression of *AaeAPL1* was slightly different to observations of *AaeLRIM1*, with the highest levels observed for 72-hours and 240-hours post infection for Key West and Orlando *Ae. aegypti*, respectively (Figure 4). All pairwise comparisons of treatment groups were significantly different from one another except for 3-hours, 120-hours, and 168-hours post infection (Figure 4). For comparison, the amount of CHIKV imbibed by mosquitoes (3-hours) and the viral titer at 72-hours, 168-hours, and 240-hours days post infection are described in Table 3B. 

To determine paralogues of *AeaAPL1* expression in response to CHIKV exposure, we measured *AeaLRIM3*, *AeaLRIM4, AeaLRIM15, AeaLRIM16*, and *AeaLRIM17* expressions in *Ae. aegypti*. Similar to responses to ZIKV exposure, MANOVA showed significant effects of strain of *Ae. aegypti*, time, and their interaction (Table 5B). In all cases, gene expression of all five paralogues of *AeaAPL1* were higher for Key West than Orlando strains with *AeaLRIM3* and *AeaLRIM16* contributing far more than the other paralogues. For the time effect, *AeaLRIM3* and *AeaLRIM16* contributed far more than the other paralogues, with by far the highest expressions at 24-hours post infection. Gene expression for the remaining paralogues were similarly highest at 24-hours post infection. Gene expression for all the paralogues tended to be lowest later during the infection process (168- and 240-hour post infection).

**Figure 5:**
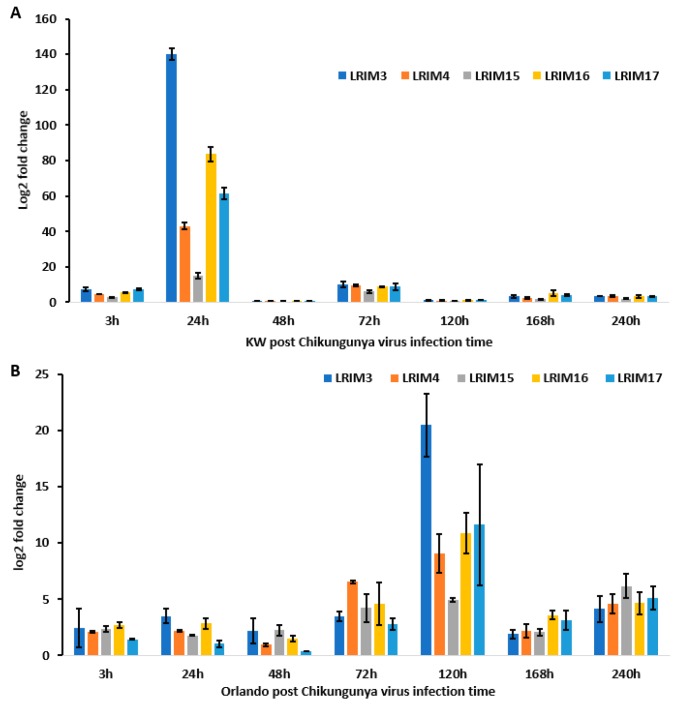
*AaeLRIM3, AaeLRIM4, AaeLRIM15, AaeLRIM16*, and *AaeLRIM17* relative expression level fold changes in *Aedes aegypti* female infected with CHIKV. The fold change was calculated using the 2 *^[−average ΔΔCT]^* method. *Δ*Ct (Control) = Ct (*AaeLRIM1*/*AaeAPL1*) − Ct (*AeaActin*); *Δ*Ct (infected-CHIKV) = Ct (*AaeLRIM1*/*AaeAPL1*) − Ct (*AeaActin*); *ΔΔ*Ct =ΔCt (infected-CHIKV) − *Δ*Ct (Control). The 3, 24, 48, 72, 120, 168, and 240 h represented gene expression post infection with CHIKV. (**A**) KW strain female *Ae. aegypti*; (**B**) Orlando strain female *Ae. aegypti*.

For the interaction, SCCs showed that *AeaLRIM3* contributed 2- to 76-fold greater than the other paralogues. As previously described, we made comparisons between strains and held time constant. Gene expression for *AeaLRIM3* was significantly higher at 3-, 24-, and 72-hours post infection for Key West than Orlando strains of *Ae. aegypti*. In contrast, gene expression for *AeaLRIM3* was significantly higher at 120-hours post infection for Orlando than Key West strains of *Ae. aegypti* (Figure 5A,B). No other comparisons were significantly different from one another. 

## 3. Discussion

Identification and characterization of genes related to *LRIM1* and *APL1C* revealed novel innate immune factors and furthered our understanding of their presumed molecular functions. Waterhouse et al. (2010) used comparative sequenced genomes: *An. gambiae*, *Ae. aegypti*, and *Cx. quinquefasciatus* revealed that mosquito LRIM proteins can be classified into four distinct subfamilies by a variable number of LRRs [16]. Our phylogenetic tree of paralogue showed that *AaeAPL1* (or *AaeLRIM2*) sequence-structure-function was most closely related to *AaeLRIM4*.

We analyzed developmental changes in the gene expression of LRR-containing proteins in *Ae. aegypti* eggs, larvae, pupae, and adults. The nucleus gene LRR-containing proteins, *AaeLRIM1, AaeAPL1*, and the other five paralogues are expressed <100-fold lower than the other nuclear genes, such as defensin, during all developmental stages examined [40]. Our data show that the expression of *AaeLRIM1* (AAEL012086), *AaeAPL1* (AAEL009520-RA), and the other five paralogues is not only regulated by development, but also by the varying environmental origin (or permethrin resistant selected strain) of mosquito strains in *Ae. aegypti*. For both immature and adult stages, we observed higher expression of *AaeLRIM1* than *AaeAPL1* in Key West *Ae. aegypti* and higher expression of *AaeAPL1* than *AaeLRIM1* in Orlando *Ae. aegypti.* These differences in responses may be attributable to differences in insecticide resistance among the Orlando (permethrin susceptible) and Key West (permethrin resistant) strains of *Ae. aegypti*. However, we are unable to rule out that other differences between these two strains of *Ae. aegypti* (e.g., geographic origin and founder effects) may contribute to the observed differences in gene expressions. For the immature, but not adult stages, expression of *AaeAPL1* and *AaeLRIM1* increased with the developmental stage. The strain effect for the analyses were modified by interactions with other factors, suggesting complex interactions between gene expression and immature stage, adult age, and sex. To our knowledge, this is the first exploration of *AaeLRIM* gene expression during *Ae*. *aegypti* development. 

In *Anopheles*, two leucine-rich repeat (LRR) protein related genes, *LRIM1* and *APL1*, have been shown to strongly affect *P. berghei* development in the mosquito midgut and have been identified as major mosquito factors that regulate parasite loads [11,13,14,15,41,42,43,44]. In *Ae. aegypti*, the likely LRIM1 orthologue is upregulated with other immune genes following infection with *Wolbachia* bacteria, resulting in immune activation and shortened mosquito life spans [45]. However, there is no information available for revealing *LRIM1* and *APL1* affected by arboviruses. Our data demonstrates that gene expression of LRR-containing proteins, *AaeLRIM1* and *AaeAPL1*, not only affect regulated parasites in the *Anopheles*, but are also altered by arbovirus infection in *Ae. aegypti*. Biophysical analysis of *An. gambiae* LRR proteins, *APL1A1*, *APL1B*, and *APL1C*, can arrange an extended, flexible heterodimer with *LRIM1*, providing a repertoire of functional innate immune complexes to protect *An. gambiae* from a diverse array of pathogens [14]. Future studies are needed to identify how proteins, *AaeLRIM1* and *AaeAPL1*, may influence progression of infection of arboviruses in *Ae. aegypti*. Some of the highest changes in expression of *AaeAPL1* and *AaeLRIM1* for both strains of *Ae. aegypti* occurred 24–72 h post infection with CHIKV, which approximates the time when *Ae. aegypti* acquire disseminated infections [24]. However, this response appears to be earlier for the Key West strain (approximately 24 h), which may suggest alterations in immune responses between permethrin susceptible and resistant *Ae. aegypti* (Shin et al. unpublished data). Changes in the expression of *AaeLRIM1* and *AaeAPL1* followed a different pattern for ZIKV infected mosquitoes, suggesting gene expression changes depend on the particular arbovirus. For both strains of *Ae. aegypti*, *AaeLRIM1* tended to be highest early during infection and declined at later points. In contrast, expression of *AaeAPL1* was low early during infection and higher at later measured times. 

We also described the gene expression of five paralogues of *AaeAPL1 (AaeLRIM3, AaeLRIM4, AaeLRIM15, AaeLRIM16*, and *AaeLRIM17)* in *Ae. aegypti* following ingestion of arbovirus infected blood. The relative contributions of the five paralogues of *AaeAPL1* differed in response to infection with either ZIKV or CHIKV, which may suggest differences in mounting an immune response between viruses of different families. Gene expression of *AeaLRIM16* and *AeaLRIM17* made the largest contributions to significant treatment effects for mosquitoes orally exposed to ZIKV. Along the same lines, *AeaLRIM3* and *AeaLRIM16* made the largest contributions to significant treatment effects for mosquitoes following oral ingestion of CHIKV infected blood. However, there were some common features observed in the gene expression of the paralogues of *AaeAPL1.* Gene expression of all five paralogues of *AaeAPL1* were usually the highest within the first 48-hours following ingestion of ZIKV or CHIKV infected blood, with lower rates of expression later during the infection process.

Studying the immune system of mosquitoes provides insights into significant opportunities to bridge tissue damage, immune invasions mechanisms, and the immune response against pathogens [4]. More importantly, unveiling a newfound understanding of mosquito immunity will shed light on the fight against disease-spreading pathogens, including ZIKV and CHIKV. Understanding the mechanisms that allow pathogens to grow and replicate in mosquitoes will provide insights into the mechanisms of mosquito-pathogen interactions. Finding exact immune evasion strategies of pathogens will help produce novel strategies that are effective at controlling them. 

## 4. Materials and Methods

### 4.1. Mosquito Strains and Developmental Stages of Aedes aegypti

*Ae. aegypti* larvae were collected from Key West (24.55°N, 81.78°W), Florida, USA since 2011 and were initially tested for permethrin resistance, then subjected to permethrin selection for 15 generations and again assayed for resistance strain [34]. Key West strain *Ae. aegypti*, referred to as the resistant strain, was maintained at the Florida Medical Entomology Laboratory (FMEL) in Vero Beach, FL, USA. The Orlando population of *Ae. aegypti* was collected from Orlando, FL, USA and reared in the Mosquito and Fly Research Unit, Center for Medical, Agricultural and Veterinary Entomology, ARS-USDA in Gainesville, FL since 1952. The Orlando strain is recognized as a permethrin susceptible strain of *Ae. aegypti* [46]. For the experiments, mosquito eggs were hatched and reared in a rearing chamber at 27 °C [40]. We sampled eggs, larvae, pupae, and adults to measure developmentally regulated gene expression of Leucine-Rich Repeat Proteins in *Ae. aegypti*. We collected 100 µg of eggs, 20 larvae at the third instar stage, 20 pupae, 10 adult mosquitoes, 10 males and 10 females at 1-day-old (teneral), 3-day-old, 5-day-old, 7-day-old, and 10-day-old adults. The experiments were repeated three times.

### 4.2. Chikungunya Virus and Zika Virus Infection

Four-day-old female adults were fed blood containing either CHIKV, ZIKV, or blood without virus as the control [34,40]. Isolates of the Indian Ocean lineage of CHIKV (LR2006-OPY1, GenBank accession: KT449801) from Réunion and the Asian lineage of ZIKV (strain PRVABC59, GenBank accession # KU501215.1) from Puerto Rico were cultured in African green monkey (Vero) cells and used in the mosquito infection study. Ten mosquitos were used in each sample and three independent experiments were conducted for each time point. The detailed procedures were described in the previous publication [34,40]. 

Arboviruses were propagated on monolayers of African green monkey kidney (Vero) cells at a multiplicity of infection of 0.1 and environmental conditions of 37 °C and 5% CO_2_. Monolayers of cells were covered with 24 mL media (M199 medium supplemented with 10% fetal bovine serum, penicillin/streptomycin, and mycostatin). For CHIKV and ZIKV, we collected the media with virus on days three and six, respectively, and mixed it with defibrinated bovine blood containing 0.005 M ATP. Mosquitoes were allowed to feed on infected blood for one hour feeding trials using a Hemotek membrane feeding system. Samples of blood were taken during feeding trials to allow for quantification of the amount of virus presented to mosquitoes. We also allowed mosquitoes to feed on a blood and media mixture without the presence of arboviruses (controls). Mosquitoes were fed 8.0-8.3 log10 pfu/mL of CHIKV for each strain and 6.4 log10 pfu/mL of ZIKV. 

Following feeding trials, fully engorged mosquitoes were sorted using light microscopy (10×) (Carson, COPYRIGHT 2006 USA, UK, HK, CHINA, Made in China) and held in cages (h by d: 10 cm by 10 cm) maintained at a 12:12 h light:dark photoperiod and 30 °C. Mosquitoes were provided with an oviposition substrate and 10% sucrose solution on cotton pads. Cohorts of mosquitoes were killed and stored at −80 °C at the following sample periods after ingesting infected blood: 3-h, 12-h (ZIKV only), 24-hours, 48-hours, 72-hours, 120-hours, 168-hours, and 240-hours. Mosquitoes were deprived of sucrose, but not water 1-day before trials used to measure transmission on 3, 5, 7, and 10 days following ingestion of ZIKV and CHIKV infection blood. 

### 4.3. RNA Extraction

Samples were homogenized with a plastic pestle in 1.5 mL tubes. Total RNAs were extracted using TRIzol reagent according to the manufacturer’s instructions (Ambion, Life Technologies, Carlsbad, CA, USA) following the standard protocol [40]. The RNA samples were digested by DNase I (RNase-free), according to the manufacturer’s instructions (Thermo Scientific, Wilmington, DE, USA). The purified RNA samples were quantitated by an NANODROP 2000 Spectrophotometer (Thermo Scientific, Wilmington, DE, USA). 

### 4.4. cDNA Synthesis and qPCR Amplification

cDNAs from 2 µg of total purified RNA were synthesized using a Cloned AMV First-Strand cDNA Synthesis Kit Invitrogen™ and Oligo (dT) 20 primer, according to the manufacturer’s instructions (Invitrogen, Carlsbad, CA). The reaction was terminated by heat inactivation at 95 °C for 5 min. The cDNA samples for qPCR from the developmental stages, infected treatment, and controls were diluted by adding 80 µL ddH_2_O to 20 µl reaction solution [47]. 

The quantitative PCR (qPCR) assay for target genes, *AaeLRIM1* and *AaeAPL1*, and reference gene, *AaeActin*, in *Ae. aegypti* was achieved using a BIO-RAD C1000 Touch Thermal Cycler, CFX 96™ Real-Time System (BIO-RAD, Hercules, CA, USA). The qPCR reaction mixture with a volume of 15 µl in Multiwell Plates 96 contained 1 µl diluted cDNA, 0.5 µM primers, and 1× master mix of PowerUP SYBR^®^ Green Master Mix (Applied Biosystems, Thermo Fisher Scientific, Foster City, CA, USA). In every qPCR run, *AaeActin* was employed as an internal control to normalize for variation in the amount of cDNA template. The PCR primers for *AaeLRPIM*, and *AaeAPL1* genes were designed from the coding region based on GenBank, Accession Number using Primer3 http://primer3.ut.ee (accessed on: Jul 20, 2017) (Table 6). The qPCR thermal cycling parameters were the same as previous publication [48]. Relative expression levels were calculated as follows for the developmental stages. First, *AaeLRIM/AaeAPL1* transcript levels relative to a standard (*AaeActin*) were calculated using the formula, *Δ*CT = CT (*AaeLRIM/AaeAPL1*) − CT (*AaeActin*). Second, an average *Δ*CT value for each sample was calculated. Third, relative expression levels were calculated using the equation, 10,000 × 2 ^[−average *Δ*CT]^ [40,48]. Relative expression levels were calculated as follows for the treatment and control adults. First, *AaeLRIM1* or *AaeAPL1* transcript levels relative to a standard (*AaeActin*) were calculated using the formula, *Δ*CT = CT (*AaeLRIM1/AaeAPL1*) − CT (*AaeActin*). Then, *ΔΔ*CT= *Δ*CT (infected) − *Δ*CT (control) value for each sample was calculated. Third, relative expression levels were calculated using the equation, 1 × 2 ^[−average *ΔΔ*CT]^ [40,48,49,50,51].

### 4.5. RNA-Seq Library Sequencing, Data Mining, and RNA-seq Analysis

RNA sequencing and RNA-seq analysis were featured in a previous publication [34]. Gene expression was assessed by counting the number of mapped reads for each transcript [52]. Significantly up- and downregulated genes were determined using the adjusted *p*-value (p-adj), log2 fold-change (log2FC), or both for downstream analysis. The RNA-seq data have been deposited to NCBI (https://www.ncbi.nlm.nih.gov/gds/?term=GSE118858). RNA-seq analysis data provided 60 leucine-rich repeat related transcriptions in the *Ae. aegypti* in response to Zika virus (Table S1A–D). 

### 4.6. Statistical Analysis

For this study, we were interested in measuring the magnitude of gene expression of components of the leucine-rich repeats (LRR)-containing domain as well as the relationship between gene expressions. So, we used a multivariate analysis of variance approach (MANOVA), which enabled us to simultaneously measure various treatment effects (e.g., developmental stage, infection with ZIKV and CHIKV) on the expression of multiple genes. Further, the MANOVA is associated with standardized canonical coefficients, which describe the relative contribution of each of the variables (genes) to a treatment effect and their direction to one another (positive or negative) [53].

Multivariate analysis of variance (MANOVA) and ANOVA were used to measure developmentally regulated gene expression of *AaeLRIM1* and *AaeAPL1*. The relative contribution and relationship of *AaeLRIM1* and *AaeAPL1* to developmental treatment effects were assessed using standardized canonical coefficients (SCC) (PROC GLM, SAS 9.22). When significant effects were detected, we used univariate comparisons among treatment least-squares means for the developmental stages (Tukey-Kramer method). Separate analyses were performed for the immature stages and adult stages. Similarly, we used MANOVA to measure the expression of *AaeLRIM1* or *AaeAPL1* following ingestion of CHIKV and ZIKV infected blood. We tested for all main treatment factors and interactions.

## Figures and Tables

**Table 1:** (**A**) Female *Aedes aegypti* transcriptomic RNA-seq data show leucine-rich repeat proteins related genes significantly upregulated in the Zika infection in the Key West strain compared with the Orlando strain *Aedes aegypti* 7-days post infection (Log2FC, Log2 fold change); (**B**) female *Aedes aegypti* transcriptomic RNA-seq data show leucine-rich repeat proteins related genes significantly upregulated/downregulated in the control (uninfected blood-feeding only) in the Key West strain compared with the Orlando strain *Aedes aegypti* 7-days post injection; (**C**) female *Aedes aegypti* transcriptomic RNA-seq data show leucine-rich repeat proteins related genes significantly dysregulated in the Key West strain *Aedes aegypti* 7-days post infection with ZIKV compared with control (uninfected blood-feeding only) in the Key West strain; (**D**) female *Aedes aegypti* transcriptomic RNA-seq data show leucine-rich repeat proteins related genes significantly dysregulated in the Orlando strain *Aedes aegypti* 7-days post infection with ZIKV compared with control (uninfected blood-feeding only) in the Orlando strain.

Transcript ID	Log2FC	p-adj	Gene Description
(**A**)			
AAEL001401-RA	3.2722	2.3 × 10^−27^	leucine-rich immune protein (Short)
AAEL001402-RA	3.2947	3.3 × 10^−33^	leucine-rich immune protein (Short)
AAEL001414-RA	3.3721	2.1 × 10^−36^	leucine-rich immune protein (Short)
AAEL001417-RA	3.9484	2.1 × 10^−5^	leucine-rich immune protein (Short)
AAEL001420-RA	3.4335	1.6 × 10^−73^	leucine-rich immune protein (Short)
AAEL001649-RA	-0.2792	4.6 × 10^−3^	leucine aminopeptidase
AAEL002295-RA	2.4545	1.9 × 10^−44^	leucine-rich transmembrane protein
AAEL002615-RA	2.2527	4.9 × 10^−12^	leucine-rich transmembrane protein
AAEL003262-RA	1.8141	3.9 × 10^−5^	leucine-rich transmembrane protein
AAEL003408-RA	1.3747	5.3 × 10^−6^	leucine-rich transmembrane protein
AAEL003713-RA	1.397	7.2 × 10^−4^	leucine-rich transmembrane protein
AAEL003720-RA	1.1688	5.6 × 10^−5^	leucine-rich transmembrane protein
AAEL005762-RA	2.2277	2.7 × 10^−5^	leucine-rich transmembrane protein
AAEL006975-RA	1.7989	8.7 × 10^−5^	leucine aminopeptidase
AAEL007103-RA	3.2536	7.5 × 10^−11^	leucine-rich immune protein (TM)
AAEL009520-RA ^1^	3.0383	4.5 × 10^−25^	leucine-rich immune protein (Long)
AAEL010125-RA	3.2598	7.0 × 10^−8^	leucine-rich immune protein (Coil-less)
AAEL010128-RA	4.8149	2.4 × 10^−7^	leucine-rich immune protein (Long)
AAEL010656-RA	2.7658	3.8 × 10^−7^	leucine-rich immune protein (Short)
AAEL012086-RA	2.4679	1.7 × 10^−12^	leucine-rich immune protein (Long)
AAEL012092-RA	2.1252	2.6 × 10^−28^	leucine-rich repeat protein
AAEL012093-RA	2.2739	1.9 × 10^−10^	leucine-rich transmembrane protein
AAEL012255-RA	4.2146	1.9 × 10^−4^	leucine-rich immune protein (Short)
(**B**)			
AAEL000243-RA	5.2443	1.8 × 10^−26^	leucine-rich transmembrane protein
AAEL009894-RA	−0.9167	2.9 × 10^−4^	leucine-rich immune protein (Coil-less)
AAEL010286-RA	3.0287	1.5 × 10^−5^	leucine-rich transmembrane protein
(**C**)			
AAEL000243-RA	−6.6469	7.6 × 10^−44^	leucine-rich transmembrane protein
AAEL003408-RA	2.1477	1.8 × 10^−4^	leucine-rich transmembrane protein
AAEL009894-RA	0.8665	9.7 × 10^−8^	leucine-rich immune protein (Coil-less)
AAEL010286-RA	−3.3918	5.0 × 10^−9^	leucine-rich transmembrane protein
(**D**)			
AAEL009520-RA ^2^	−2.2074	2.8 × 10^−4^	leucine-rich immune protein (Long)
AAEL010128-RA	−5.3692	2.8 × 10^−6^	leucine-rich immune protein (Long)
AAEL001402-RA	−3.3551	6.1 × 10^−8^	leucine-rich immune protein (Short)
AAEL010656-RA	−2.5623	4.4 × 10^−3^	leucine-rich immune protein (Short)
AAEL012255-RA	−4.1624	3.1 × 10^−5^	leucine-rich immune protein (Short)

^1^ AAEL009520-RA is the same gene as AAEL024406. ^2^ AAEL009520-RA is the same gene as AAEL024406.

**Table 2:** (**A**) MANOVA results for strain and stage effects of the *AaeLRIM1* and *AaeAPL1* RNA profile in immature stages of *Ae. aegypti*. (**B**) MANOVA results for strain, sex, and age effects of the *AaeLRIM1* and *AaeAPL1* RNA profile in adult stages of *Ae. aegypti*.

Treatment	Pillai’s Trace	df (Numerator and Denominator)	*p*-value	Standardized Canonical Coefficients	
				*AaeLR1M1*	*AaeAPL1*
(**A**)					
**Strain**	0.99	2, 11	<0.0001	12.12	−10.76
**Stage**	1.99	4, 24	<0.0001	16.28	8.35
**Strain × Stage**	1.89	4, 24	<0.0001	11.09	−11.57
(**B**)					
**Strain**	0.99	2, 39	<0.0001	47.84	−7.90
**Sex**	1.00	2, 39	<0.0001	49.46	−4.80
**Age**	1.98	8, 80	<0.0001	49.48	−3.43
**Strain × Sex**	1.00	2, 39	<0.0001	48.66	−1.11
**Strain × Age**	1.93	8, 80	<0.0001	46.59	−9.05
**Sex × Age**	1.99	8, 80	<0.0001	49.49	−4.55
**Strain × Sex x Age**	1.98	8, 80	<0.0001	49.52	−4.06

**Table 3:** (**A**) Zika virus titers (log10 pfu/mL) in infectious blood meals and mosquitoes for Key West and Orlando strains of *Aedes aegypti*. (**B**) Chikungunya virus titers (log10 pfu/mL) in infectious blood meals and mosquitoes for Key West and Orlando strains of *Aedes aegypti*.

Strains	Initial Dose in Bloodmeal	Freshly Fed (3 h)	3 Days (72 h) Post Infection	7 Days (168 h) Post Infection	10 Days (240 h) Post Infection
(**A**)					
**Key West**	6.4 ± 0.09	4.30 ± 0.0	3.96 ± 0.26	4.11 ± 1.78	6.57 ± 0.05
**Orlando**	6.4 ± 0.08	4.17 ± 0.39	3.66 ± 0.27	3.58 ± 1.23	4.49 ± 0.34
(**B**)					
**Key West**	8.0 ± 0.09	5.23 ± 0.10	4.7 ± 0.81	5.4 ± 0.46	5.20 ± 0.49
**Orlando**	8.3 ± 0.08	4.56 ± 1.16	5.04 ± 0.71	5.81 ± 0.48	5.53 ± 0.33

Zika virus (strain PRVABC59, GenBank accession # KU501215.1) isolated from a human infected in Puerto Rico in 2015. Primers: ZIKV-FWD: 5′-CTTCTTATCCACAGCCGTCTC-3′; ZIKV-REV: 5′-CCAGGCTTCAACGTCGTTAT-3′. CHIKV (LaReunion strain LR2006-OPY1, GenBank KT449801) from a human infected on La Réunion Island in 2006 (Parola et al. 2006). Primers: CHIKV-FWD: 5′GTACGGAAGGTAAACTGGTATGG-3′; CHIKV-REV: 5′-TCCACCTCCCACTCCTTAAT-3′.

**Table 4:** (**A**) MANOVA results for strain and time effects of the *AaeLRIM1* and *AaeAPL1* RNA profile in response to Zika virus infection of *Ae. aegypti*. (**B**) MANOVA results for strain and time effects of paralogues of the *AaeAPL1* RNA profile in response to Zika virus infection of *Ae. aegypti*.

(A)	Treatment	Pillai’s Trace	df (Numerator and Denominator	*p*-value	Standardized Canonical Coefficients		(B)	Treatment	Pillai’s Trace	df (Num., Denom.)	*p*-value	Standardized Canonical Coefficients				
					*AaeLR1M1*	*AaeAPL1*						*AaeLR1M3*	*AaeLRIM4*	*AaeLRIM15*	*AaeLRIM16*	*AaeLRIM17*
	**Strain**	0.86	2, 31	<0.0001	8.06	−1.16		**Strain**	0.96	5, 28	<0.0001	2.78	0.14	−4.04	11.14	2.86
	**Time**	1.94	14, 64	<0.0001	8.07	−1.01		**Time**	3.19	35, 160	<0.0001	−0.05	5.94	4.41	−1.81	8.07
	**Strain × Time**	1.86	14, 64	<0.0001	2.45	5.84		**Strain × Time**	3.50	35, 160	<0.0001	2.92	2.25	−6.76	11.38	1.44

**Table 5:** (**A**) MANOVA results for strain and time effects of *AaeLRIM1* and *AaeAPL1* RNA profile in response to chikungunya virus infection of *Ae. aegypti*. (**B**) MANOVA results for strain and time effects of paralogues of *AaeAPL1* RNA profile in response to chikungunya virus infection of *Ae. aegypti*.

(A)	Treatment	Pillai’s Trace	df (Numerator and Denominator)	*p*-value	Standardized Canonical Coefficients		(B)	Treatment	Pillai’s Trace	df (Numerator and Denominator)	*p*-value	Standardized Canonical Coefficients				
					*AaeLR1M1*	*AaeAPL1*						*AaeLR1M3*	*AaeLRIM4*	*AaeLRIM15*	*AaeLRIM16*	*AaeLRIM17*
	**Strain**	0.60	2, 27	<0.0001	5.90	1.23		**Strain**	0.99	5, 24	<0.0001	25.13	−0.51	−0.28	10.46	−0.58
	**Time**	1.77	12, 56	<0.0001	4.59	2.61		**Time**	2.70	30, 140	<0.0001	25.50	−0.99	0.22	11.01	−1.32
	**Strain × Time**	1.79	12, 56	<0.0001	6.20	0.79		**Strain × Time**	2.31	30, 140	<0.0001	24.57	−0.32	0.46	10.82	−1.08

**Table 6:** Primers from *Aedes aegypti* for qPCR reaction.

Gene ID	Accession	Gene Name	Primer Name	Primer Sequence (5′–3′)
AAEL012086	XM_001662191.2	*AaeLRIM1*	AaeLRIM1-086-1011F	TGACAACCGGGTTAAGGAAG
			AaeLRIM1-086-1198R	TGGCCAAATCATTGTTCTCA
AAEL024406 ^1^	*XM_021843329.1*	*AaeAPL1*	AaeAPL1-406-115F	TCAACCCAGCCTCCAGATAC
			AaeAPL1-406-275R	TCAGCAGTTTCACCACTTGC
AAEL010132	XM_001660603.2	*AaeLRIM3*	AaeLRIM3-132-166F	TGTAGCCCGCAATAATCACA
			AaeLRIM3-132-405R	CTGAAGTGCTCCGTTGAACA
AAEL010128	XM_001660601.2	*AaeLRIM4*	AaeLRIM4-128-612F	TGTAGCCCGCAATAATCACA
			AaeLRIM4-128-830R	GCCAGATTAAGCTCCACGAG
AAEL007103	XM_001658083.2	*AaeLRIM15*	AaeLRIM15-103-1522F	ATGGTATTGCGTGGAGGAAG
			AaeLRIM15-103-1676R	ATCCTATCAACCGCCCTTCT
AAEL008658	XM_021855914.1	*AaeLRIM16*	AaeLRIM16-658-299F	ACACCTTCGAGAAAGCGAAA
			AaeLRIM16-658-541R	TCAACATGGGCAAATGAGAA
AAEL010125	XM_001660604.2	*AaeLRIM17*	AaeLRIM17-125-555F	GCAGTACAATTCGCTGACCA
			AaeLRIM17-125-718R	CCTTAAGCCGATTGAAGCTG
AAEL011197	XM_001655125.2	*AaeActin*	AaeActin-197-152F	AGGACTCGTACGTCGGTGAC
			AaeActin-197-590R	CGTTCAGTCAGGATCTTC

^1^ AAEL024406 is the same gene as AAEL009520-RA.

## Supplementary Materials

Supplementary materials can be found at http://www.mdpi.com/1422-0067/20/3/615/s1. Table S1A–D: Female *Aedes aegypti* transcriptomic RNA-seq data show Leucine-Rich Repeat Proteins 7-days post infection or post injection between Key west and Orlando strains. Figure S1. Evolutionary analysis of paralogues of *AeaAPL1* of the *Ae. Aegypti*. Figure S2A–E. *AaeLRIM3, AaeLRIM4, AaeLRIM15, AaeLRIM16*, and *AaeLRIM17* relative expression in the all developmental stages.

## Abbreviations

ZIKV	Zika Virus
CHIKV	Chikungunya Virus
LRR	Leucine-Rich Repeats
LRIM1	Leucine-Rich Repeat Immune Protein 1
APL1	Anopheles Plasmodium-responsive Leucine-rich Repeat 1

## References

[1] Ng A., Xavier R.J. (2011). Leucine-rich repeat (LRR) proteins: Integrators of pattern recognition and signaling in immunity. Autophagy.

[2] Ausubel F.M. (2005). Are innate immune signaling pathways in plants and animals conserved?. Nat. Immunol..

[3] Medzhitov R. (2007). Recognition of microorganisms and activation of the immune response. Nature.

[4] Kumar A., Srivastava P., Sirisena P., Dubey S.K., Kumar R., Shrinet J., Sunil S. (2018). Mosquito Innate Immunity. Insects.

[5] Kantor A.M., Dong S., Held N.L., Ishimwe E., Passarelli A.L., Clem R.J., Franz A.W. (2017). Identification and initial characterization of matrix metalloproteinases in the yellow fever mosquito, *Aedes aegypti*. Insect. Mol. Biol..

[6] Dong S., Kantor A.M., Lin J., Passarelli A.L., Clem R.J., Franz A.W. (2016). Infection pattern and transmission potential of chikungunya virus in two New World laboratory-adapted *Aedes aegypti* strains. Sci. Rep..

[7] Houk E.J., Hardy J.L., Chiles R.E. (1981). Permeability of the midgut basal lamina in the mosquito, *Culex tarsalis* Coquillett (Insecta, Diptera). Acta Trop..

[8] Passarelli A.L. (2011). Barriers to success: How baculoviruses establish efficient systemic infections. Virology.

[9] Bartholomay L.C., Michel K. (2018). Mosquito Immunobiology: The Intersection of Vector Health and Vector Competence. Annu. Rev. Entomol..

[10] Bartholomay L.C., Cho W.L., Rocheleau T.A., Boyle J.P., Beck E.T., Fuchs J.F., Liss P., Rusch M., Butler K.M., Wu R.C. (2004). Description of the transcriptomes of immune response-activated hemocytes from the mosquito vectors *Aedes aegypti* and *Armigeres subalbatus*. Infect. Immunity.

[11] Upton L.M., Povelones M., Christophides G.K. (2015). *Anopheles gambiae* blood feeding initiates an anticipatory defense response to *Plasmodium berghei*. J. Innate Immunity.

[12] Habtewold T., Povelones M., Blagborough A.M., Christophides G.K. (2008). Transmission blocking immunity in the malaria non-vector mosquito *Anopheles quadriannulatus* species A. PLoS Pathog..

[13] Povelones M., Waterhouse R.M., Kafatos F.C., Christophides G.K. (2009). Leucine-rich repeat protein complex activates mosquito complement in defense against *Plasmodium* parasites. Science.

[14] Williams M., Summers B.J., Baxter R.H. (2015). Biophysical analysis of *Anopheles gambiae* leucine-rich repeat proteins APL1A1, APL1B [corrected] and APL1C and their interaction with LRIM1. PLoS ONE.

[15] Fraiture M., Baxter R.H., Steinert S., Chelliah Y., Frolet C., Quispe-Tintaya W., Hoffmann J.A., Blandin S.A., Levashina E.A. (2009). Two mosquito LRR proteins function as complement control factors in the TEP1-mediated killing of Plasmodium. Cell Host Microbe.

[16] Waterhouse R.M., Povelones M., Christophides G.K. (2010). Sequence-structure-function relations of the mosquito leucine-rich repeat immune proteins. BMC Genomics.

[17] Riehle M.M., Xu J., Lazzaro B.P., Rottschaefer S.M., Coulibaly B., Sacko M., Niare O., Morlais I., Traore S.F., Vernick K.D. (2008). *Anopheles gambiae* APL1 is a family of variable LRR proteins required for Rel1-mediated protection from the malaria parasite, *Plasmodium berghei*. PLoS ONE.

[18] Blandin S.A., Wang-Sattler R., Lamacchia M., Gagneur J., Lycett G., Ning Y., Levashina E.A., Steinmetz L.M. (2009). Dissecting the genetic basis of resistance to malaria parasites in *Anopheles gambiae*. Science.

[19] Kwon H., Arends B.R., Smith R.C. (2017). Late-phase immune responses limiting oocyst survival are independent of TEP1 function yet display strain specific differences in *Anopheles gambiae*. Parasit Vectors.

[20] Smith R.C., Barillas-Mury C. (2016). *Plasmodium* Oocysts: Overlooked Targets of Mosquito Immunity. Trends Parasitol..

[21] Tsetsarkin K.A., Chen R., Weaver S.C. (2016). Interspecies transmission and chikungunya virus emergence. Curr. Opin. Virol..

[22] Lanciotti R.S., Valadere A.M. (2014). Transcontinental movement of Asian genotype chikungunya virus. Emerg. Infect. Dis..

[23] Leparc-Goffart I., Nougairede A., Cassadou S., Prat C., de Lamballerie X. (2014). Chikungunya in the Americas. Lancet.

[24] Alto B.W., Wiggins K., Eastmond B., Velez D., Lounibos L.P., Lord C.C. (2017). Transmission risk of two chikungunya lineages by invasive mosquito vectors from Florida and the Dominican Republic. PLoS Negl. Trop. Dis..

[25] Powers A.M. (2016). How Chikungunya Virus Virology Affects Its Epidemiology and Transmission: Implications for Influencing Public Health. J. Infect. Dis..

[26] Caglioti C., Lalle E., Castilletti C., Carletti F., Capobianchi M.R., Bordi L. (2013). Chikungunya virus infection: An overview. New Microbiol..

[27] Gasque P., Bandjee M.C., Reyes M.M., Viasus D. (2016). Chikungunya Pathogenesis: From the Clinics to the Bench. J. Infect. Dis..

[28] Solomon T., Baylis M., Brown D. (2016). Zika virus and neurological disease--approaches to the unknown. Lancet Infect. Dis..

[29] Lanciotti R.S., Lambert A.J., Holodniy M., Saavedra S., Signor L.e.C. (2016). Phylogeny of Zika Virus in Western Hemisphere, 2015. Emerg. Infect. Dis..

[30] Sacramento C.Q., de Melo G.R., de Freitas C.S., Rocha N., Hoelz L.V., Miranda M., Fintelman-Rodrigues N., Marttorelli A., Ferreira A.C., Barbosa-Lima G. (2017). The clinically approved antiviral drug sofosbuvir inhibits Zika virus replication. Sci. Rep..

[31] Cuevas E.L., Tong V.T., Rozo N., Valencia D., Pacheco O., Gilboa S.M., Mercado M., Renquist C.M., González M., Ailes E.C. (2016). Preliminary Report of Microcephaly Potentially Associated with Zika Virus Infection During Pregnancy—Colombia, January–November 2016. MMWR Morb. Mortal. Wkly. Rep..

[32] Pinto-Díaz C.A., Rodríguez Y., Monsalve D.M., Acosta-Ampudia Y., Molano-González N., Anaya J.M., Ramírez-Santana C. (2017). Autoimmunity in Guillain-Barré syndrome associated with Zika virus infection and beyond. Autoimmunity Rev..

[33] Dong S., Behura S.K., Franz A.W.E. (2017). The midgut transcriptome of *Aedes aegypti* fed with saline or protein meals containing chikungunya virus reveals genes potentially involved in viral midgut escape. BMC Genomics.

[34] Zhao L., Alto B.W., Shin D., Yu F. (2018). The Effect of Permethrin Resistance on *Aedes aegypti* Transcriptome Following Ingestion of Zika Virus Infected Blood. Viruses.

[35] Etebari K., Hegde S., Saldaña M.A., Widen S.G., Wood T.G., Asgari S., Hughes G.L. (2017). Global Transcriptome Analysis of *Aedes aegypti* Mosquitoes in Response to Zika Virus Infection. mSphere.

[36] Shrinet J., Srivastava P., Sunil S. (2017). Transcriptome analysis of Aedes aegypti in response to mono-infections and co-infections of dengue virus-2 and chikungunya virus. Biochem. Biophys. Res. Commun..

[37] Angleró-Rodríguez Y.I., MacLeod H.J., Kang S., Carlson J.S., Jupatanakul N., Dimopoulos G. (2017). Molecular Responses to Zika Virus: Modulation of Infection by the Toll and Jak/Stat Immune Pathways and Virus Host Factors. Front. Microbiol..

[38] Kumar S., Stecher G., Tamura K. (2016). MEGA7: Molecular Evolutionary Genetics Analysis Version 7.0 for Bigger Datasets. Mol. Biol. Evol..

[39] Smith L.B., Kasai S., Scott J.G. (2016). Pyrethroid resistance in *Aedes aegypti* and *Aedes albopictus*: Important mosquito vectors of human diseases. Pestic Biochem. Physiol..

[40] Zhao L., Alto B.W., Smartt C.T., Shin D. (2017). Transcription Profiling for Defensins of *Aedes aegypti (*Diptera: Culicidae) During Development and in Response to Infection with Chikungunya and Zika Viruses. J. Med. Entomol..

[41] Riehle M.M., Markianos K., Niaré O., Xu J., Li J., Touré A.M., Podiougou B., Oduol F., Diawara S., Diallo M. (2006). Natural malaria infection in *Anopheles gambiae* is regulated by a single genomic control region. Science.

[42] Osta M.A., Christophides G.K., Kafatos F.C. (2004). Effects of mosquito genes on *Plasmodium* development. Science.

[43] Osta M.A., Christophides G.K., Vlachou D., Kafatos F.C. (2004). Innate immunity in the malaria vector *Anopheles gambiae*: Comparative and functional genomics. J. Exp. Biol..

[44] Blandin S., Shiao S.H., Moita L.F., Janse C.J., Waters A.P., Kafatos F.C., Levashina E.A. (2004). Complement-like protein TEP1 is a determinant of vectorial capacity in the malaria vector *Anopheles gambiae*. Cell.

[45] Kambris Z., Cook P.E., Phuc H.K., Sinkins S.P. (2009). Immune activation by life-shortening *Wolbachia* and reduced filarial competence in mosquitoes. Science.

[46] Estep A.S., Sanscrainte N.D., Waits C.M., Louton J.E., Becnel J.J. (2017). Resistance Status and Resistance Mechanisms in a Strain of *Aedes aegypti* (Diptera: Culicidae) From Puerto Rico. J. Med. Entomol..

[47] Zhao L., Alto B.W., Duguma D. (2017). Transcriptional Profile for Detoxification Enzymes AeaGGT1 and AaeGGT2 From *Aedes aegypti* (Diptera: Culicidae) in Response to Larvicides. J. Med. Entomol..

[48] Zhao L., Pridgeon J.W., Becnel J.J., Clark G.G., Linthicum K.J. (2008). Cytochrome c gene and protein expression: Developmental regulation, environmental response, and pesticide sensitivity in *Aedes aegypti*. J. Med. Entomol..

[49] Portereiko M.F., Sandaklie-Nikolova L., Lloyd A., Dever C.A., Otsuga D., Drews G.N. (2006). NUCLEAR FUSION DEFECTIVE1 encodes the *Arabidopsis* RPL21M protein and is required for karyogamy during female gametophyte development and fertilization. Plant Physiol..

[50] Livak K.J., Schmittgen T.D. (2001). Analysis of relative gene expression data using real-time quantitative PCR and the 2(-Delta Delta C(T)) Method. Methods.

[51] Portereiko M.F., Lloyd A., Steffen J.G., Punwani J.A., Otsuga D., Drews G.N. (2006). AGL80 is required for central cell and endosperm development in *Arabidopsis*. Plant Cell.

[52] Yao J.Q., Yu F. (2011). DEB: A web interface for RNA-seq digital gene expression analysis. Bioinformation.

[53] Scheiner S.M., Scheiner S.M., Gurevitch J. (1993). Multiple response variables and multispecies interactions. Design and Analysis of Ecological Experiments.

