# Supplementary material for: Transcriptional Profile of Aedes aegypti Leucine-Rich Repeat Proteins in Response to Zika and Chikungunya Viruses
Source: Int J Mol Sci. 2019 Jan 31;20(3):615. doi: 10.3390/ijms20030615 (PMC6386990; doi:10.3390/ijms20030615)
Supplement: Supplementary file 1 [file ijms-20-00615-s001.pdf]

Supplementary Materials: Table S1: Female *Aedes aegypti* transcriptomic RNA-seq data show Leucine-Rich Repeat Proteins 7-days post infection or post injection between Key west and Orlando strains.

Table 1. Female *Aedes aegypti* transcriptomic RNA-seq data show Leucine-Rich Repeat Proteins related genes expression in the Zika infection in Key West strain compared with Orlando strain *Aedes aegypti* 7-days post infection.

Transcript ID	Log2FC	p-adj	Gene description
AAEL000108-RA	0.8163	8.6×10^{-1}	leucine aminopeptidase
AAEL000243-RA	-2.1351	1.3×10^{-2}	leucine-rich transmembrane protein
AAEL000424-RA	-0.1229	1.0×10^{-1}	leucine aminopeptidase
AAEL000925-RA	-0.0806	5.5×10^{-1}	leucine-zipper-like transcriptional regulator
AAEL001401-RA	3.2722	2.3×10^{-27}	leucine-rich immune protein (Short)
AAEL001402-RA	3.2947	3.3×10^{-33}	leucine-rich immune protein (Short)
AAEL001414-RA	3.3722	2.1×10^{-36}	leucine-rich immune protein (Short)
AAEL001417-RA	3.9485	2.0×10^{-5}	leucine-rich immune protein (Short)
AAEL001420-RA	3.4335	1.6×10^{-73}	leucine-rich immune protein (Short)
AAEL001649-RA	-0.2792	4.7×10^{-3}	leucine aminopeptidase
AAEL001766-RA	1.7007	2.4×10^{-2}	leucine-rich transmembrane protein
AAEL002166-RA	-0.0764	4.8×10^{-1}	leucine rich repeat (in flii) interacting protein
AAEL002295-RA	2.4545	1.9×10^{-44}	leucine-rich transmembrane protein
AAEL002307-RA	1.0570	6.1×10^{-2}	leucine-rich transmembrane protein
AAEL002615-RA	2.2527	5.0×10^{-12}	leucine-rich transmembrane protein
AAEL003262-RA	1.8141	3.9×10^{-5}	leucine-rich transmembrane protein
AAEL003408-RA	1.3747	5.3×10^{-6}	leucine-rich transmembrane protein
AAEL003554-RA	0.0380	8.3×10^{-1}	leucine rich repeat protein
AAEL003597-RA	1.8100	2.7×10^{-1}	leucine-rich transmembrane protein
AAEL003597-RB	1.4073	4.9×10^{-1}	leucine-rich transmembrane protein
AAEL003713-RA	1.3970	7.3×10^{-4}	leucine-rich transmembrane protein
AAEL003720-RA	1.1688	5.6×10^{-5}	leucine-rich transmembrane protein
AAEL003859-RA	0.2431	2.1×10^{-1}	leucine-rich transmembrane protein
AAEL004466-RA	2.5519	4.4×10^{-1}	leucine-rich immune protein (Coil-less)
AAEL004773-RA	-0.0661	6.3×10^{-1}	leucine carboxyl methyltransferase
AAEL005351-RA	-0.0974	2.1×10^{-1}	leucine-rich transmembrane protein
AAEL005734-RA	0.0613	6.7×10^{-1}	leucine-rich transmembrane protein
AAEL005762-RA	2.2277	2.7×10^{-5}	leucine-rich transmembrane protein
AAEL006026-RA	-0.0266	9.2×10^{-1}	leucine rich protein, putative
AAEL006377-RA	1.5263	1.2×10^{-1}	leucine-rich immune protein (Coil-less)
AAEL006975-RA	1.7989	8.7×10^{-5}	leucine aminopeptidase
AAEL007103-RA	3.2536	7.5×10^{-11}	leucine-rich immune protein TM
AAEL007224-RA	1.1275	1.4×10^{-1}	leucine-rich immune protein (Coil-less)
AAEL007231-RA	0.1956	1.0×10^{-0}	leucine-rich immune protein (Coil-less)
AAEL007363-RA	0.8814	5.2×10^{-6}	leucine-rich transmembrane protein
AAEL007565-RA	-0.0052	1.0×10^{-0}	leucine rich protein, putative
AAEL007778-RA	1.5531	8.6×10^{-15}	leucine-rich transmembrane protein
AAEL007785-RA	1.0229	1.2×10^{-1}	leucine-rich transmembrane protein
AAEL008658-RA	2.4764	1.6×10^{-11}	leucine-rich immune protein TM
AAEL009520-RA[1]	3.0383	4.5×10^{-25}	leucine-rich immune protein (Long)
AAEL009792-RA	0.6257	8.4×10^{-1}	leucine-rich immune protein (Coil-less)

Transcript ID	Log2FC	p-adj	Gene description
AAEL009894-RA	-0.1055	4.2×10^{-1}	leucine-rich immune protein (Coil-less)
AAEL010111-RA	0.7172	2.1×10^{-1}	leucine-rich transmembrane protein
AAEL010125-RA	3.2598	7.0×10^{-8}	leucine-rich immune protein (Coil-less)
AAEL010128-RA	4.8149	2.4×10^{-7}	leucine-rich immune protein (Long)
AAEL010132-RA	0.1733	1.0×10^{-0}	leucine-rich immune protein (Long)
AAEL010286-RA	1.5368	1.1×10^{-1}	leucine-rich transmembrane protein
AAEL010656-RA	2.7658	3.9×10^{-7}	leucine-rich immune protein (Short)
AAEL010772-RA	0.3692	8.7×10^{-1}	Leucine-rich repeat-containing protein 50 homolog
AAEL011387-RA	0.2131	5.3×10^{-1}	leucine-rich repeat protein
AAEL011760-RA	-0.2186	8.7×10^{-1}	leucine-rich transmembrane protein
AAEL012086-RA	2.4679	1.7×10^{-12}	leucine-rich immune protein (Long)
AAEL012092-RA	2.1252	2.6×10^{-28}	leucine rich repeat protein
AAEL012093-RA	2.2739	1.9×10^{-10}	leucine-rich transmembrane protein
AAEL012255-RA	4.2146	2.0×10^{-4}	leucine-rich immune protein (Short)
AAEL012763-RA	0.2781	7.3×10^{-1}	leucine-rich immune protein (Coil-less)
AAEL012767-RA	0.2458	9.9×10^{-1}	leucine-rich immune protein (Short)
AAEL012771-RA	-2.1327	9.0×10^{-2}	leucine-rich immune protein (Coil-less)
AAEL012911-RA	0.4919	1.8×10^{-1}	leucine-rich immune protein (Coil-less)

[1] AAEL009520-RA is the same gene as AAEL024406.

Table S1B. Female *Aedes aegypti* transcriptomic RNA-seq data show Leucine-Rich Repeat Proteins related genes expression in Control (uninfected blood-feeding only) in the Key West strain compared with Orlando strain *Aedes aegypti* 7-days post injection.

Transcript ID	Log2FC	p-adj	Gene description
AAEL000108-RA	0.2407	1.0×10^{-0}	leucine aminopeptidase
AAEL000243-RA	5.2443	1.8×10^{-26}	leucine-rich transmembrane protein
AAEL000424-RA	0.2572	5.3×10^{-1}	leucine aminopeptidase
AAEL000762-RA	1.7648	4.0×10^{-1}	leucine-rich immune protein (Coil-less)
AAEL000925-RA	-0.0274	1.0×10^{-0}	leucine-zipper-like transcriptional regulator
AAEL001401-RA	1.2776	7.7×10^{-1}	leucine-rich immune protein (Short)
AAEL001402-RA	0.7638	8.5×10^{-1}	leucine-rich immune protein (Short)
AAEL001414-RA	1.1630	7.5×10^{-1}	leucine-rich immune protein (Short)
AAEL001417-RA	1.1400	5.8×10^{-1}	leucine-rich immune protein (Short)
AAEL001420-RA	0.9209	8.7×10^{-1}	leucine-rich immune protein (Short)
AAEL001649-RA	0.1852	7.8×10^{-1}	leucine aminopeptidase
AAEL001766-RA	-0.2723	9.8×10^{-1}	leucine-rich transmembrane protein
AAEL002166-RA	0.0549	9.8×10^{-1}	leucine rich repeat (in flii) interacting protein
AAEL002295-RA	-0.0871	1.0×10^{-0}	leucine-rich transmembrane protein
AAEL002307-RA	-1.9783	5.3×10^{-2}	leucine-rich transmembrane protein
AAEL002615-RA	0.1515	1.0×10^{-0}	leucine-rich transmembrane protein
AAEL003262-RA	1.5448	1.4×10^{-1}	leucine-rich transmembrane protein
AAEL003408-RA	-1.3019	2.4×10^{-1}	leucine-rich transmembrane protein
AAEL003554-RA	-0.0706	9.7×10^{-1}	leucine rich repeat protein
**Transcript ID**	**Log2FC**	**p-adj**	**Gene description**

AAEL003597-RA	0.1151	1.0×10^{-0}	leucine-rich transmembrane protein
AAEL003713-RA	0.1831	1.0×10^{-0}	leucine-rich transmembrane protein
AAEL003720-RA	-0.8548	5.4×10^{-1}	leucine-rich transmembrane protein
AAEL003859-RA	0.3130	7.8×10^{-1}	leucine-rich transmembrane protein
AAEL004466-RA	2.0123	8.8×10^{-1}	leucine-rich immune protein (Coil-less)
AAEL004773-RA	0.0049	1.0×10^{-0}	leucine carboxyl methyltransferase
AAEL005351-RA	-0.1827	5.8×10^{-1}	leucine-rich transmembrane protein
AAEL005734-RA	-0.2283	6.6×10^{-1}	leucine-rich transmembrane protein
AAEL005762-RA	-0.4492	9.2×10^{-1}	leucine-rich transmembrane protein
AAEL006026-RA	0.0909	9.6×10^{-1}	leucine rich protein, putative
AAEL006377-RA	0.2883	1.0×10^{-0}	leucine-rich immune protein (Coil-less)
AAEL006975-RA	-1.4523	2.8×10^{-1}	leucine aminopeptidase
AAEL007103-RA	1.5062	6.7×10^{-1}	leucine-rich immune protein TM
AAEL007224-RA	1.6625	6.4×10^{-1}	leucine-rich immune protein (Coil-less)
AAEL007231-RA	-0.2972	1.0×10^{-0}	leucine-rich immune protein (Coil-less)
AAEL007363-RA	-0.3259	8.7×10^{-1}	leucine-rich transmembrane protein
AAEL007565-RA	-0.0600	9.8×10^{-1}	leucine rich protein, putative
AAEL007778-RA	1.1993	5.6×10^{-1}	leucine-rich transmembrane protein
AAEL007785-RA	-0.4638	9.3×10^{-1}	leucine-rich transmembrane protein
AAEL008658-RA	0.3448	9.8×10^{-1}	leucine-rich immune protein TM
AAEL009520-RA[1]	1.7824	6.0×10^{-1}	leucine-rich immune protein (Long)
AAEL009792-RA	-0.0468	1.0×10^{-0}	leucine-rich immune protein (Coil-less)
AAEL009894-RA	-0.9167	2.9×10^{-4}	leucine-rich immune protein (Coil-less)
AAEL010111-RA	0.5586	8.8×10^{-1}	leucine-rich transmembrane protein
AAEL010125-RA	1.6477	5.3×10^{-1}	leucine-rich immune protein (Coil-less)
AAEL010128-RA	-0.4969	9.1×10^{-1}	leucine-rich immune protein (Long)
AAEL010132-RA	1.1041	6.9×10^{-1}	leucine-rich immune protein (Long)
AAEL010286-RA	3.0287	1.5×10^{-5}	leucine-rich transmembrane protein
AAEL010656-RA	-0.0488	1.0×10^{-0}	leucine-rich immune protein (Short)
AAEL010772-RA	-0.0798	1.0×10^{-0}	Leucine-rich repeat-containing protein 50 homolog
AAEL011387-RA	0.1003	1.0×10^{-0}	leucine-rich repeat protein
AAEL011760-RA	2.0781	8.8×10^{-2}	leucine-rich transmembrane protein
AAEL012086-RA	2.1793	4.9×10^{-1}	leucine-rich immune protein (Long)
AAEL012092-RA	-0.2727	9.7×10^{-1}	leucine rich repeat protein
AAEL012093-RA	-0.5269	7.5×10^{-1}	leucine-rich transmembrane protein
AAEL012255-RA	0.5067	9.4×10^{-1}	leucine-rich immune protein (Short)
AAEL012538-RA	1.3374	7.5×10^{-1}	leucine-rich immune protein (Short)
AAEL012763-RA	0.2824	9.6×10^{-1}	leucine-rich immune protein (Coil-less)
AAEL012767-RA	2.3014	1.8×10^{-1}	leucine-rich immune protein (Short)
AAEL012771-RA	2.5966	3.0×10^{-1}	leucine-rich immune protein (Coil-less)
AAEL012911-RA	-0.6582	7.1×10^{-1}	leucine-rich immune protein (Coil-less)
AAEL015627-RA	-0.1674	1.0×10^{-1}	leucine-rich immune protein (Coil-less)

[1] AAEL009520-RA is the same gene as AAEL024406.

Table S1C. Female *Aedes aegypti* transcriptomic RNA-seq data show Leucine-Rich Repeat Proteins related genes dysregulated in the Key West strain *Aedes aegypti* 7-days post infection with ZIKV compared with Control (uninfected blood-feeding only) in Key West strain.

Transcript ID	Log2FC	p-adj	Gene description
AAEL000108-RA	-1.7731	4.8×10^{-1}	leucine aminopeptidase
AAEL000243-RA	-6.6469	7.6×10^{-44}	leucine-rich transmembrane protein
AAEL000424-RA	-0.3141	3.1×10^{-1}	leucine aminopeptidase
AAEL000762-RA	-1.1738	5.4×10^{-1}	leucine-rich immune protein (Coil-less)
AAEL000925-RA	-0.0007	1.0×10^{-0}	leucine-zipper-like transcriptional regulator
AAEL001401-RA	-1.3103	6.4×10^{-1}	leucine-rich immune protein (Short)
AAEL001402-RA	-0.8166	7.5×10^{-1}	leucine-rich immune protein (Short)
AAEL001414-RA	-1.4239	5.2×10^{-1}	leucine-rich immune protein (Short)
AAEL001417-RA	-1.5243	1.7×10^{-1}	leucine-rich immune protein (Short)
AAEL001420-RA	-1.3793	5.7×10^{-1}	leucine-rich immune protein (Short)
AAEL001649-RA	-0.2873	4.6×10^{-1}	leucine aminopeptidase
AAEL001766-RA	0.1960	9.8×10^{-1}	leucine-rich transmembrane protein
AAEL002166-RA	-0.0592	9.5×10^{-1}	leucine rich repeat (in flii) interacting protein
AAEL002295-RA	0.3188	7.8×10^{-1}	leucine-rich transmembrane protein
AAEL002307-RA	1.3559	2.1×10^{-1}	leucine-rich transmembrane protein
AAEL002615-RA	0.1694	9.9×10^{-1}	leucine-rich transmembrane protein
AAEL003262-RA	-0.1092	1.0×10^{-0}	leucine-rich transmembrane protein
AAEL003408-RA	2.1477	1.8×10^{-4}	leucine-rich transmembrane protein
AAEL003554-RA	0.1223	7.6×10^{-1}	leucine rich repeat protein
AAEL003597-RB	-0.1468	1.0×10^{-0}	leucine-rich transmembrane protein
AAEL003713-RA	-0.0746	1.0×10^{-0}	leucine-rich transmembrane protein
AAEL003720-RA	0.8861	9.8×10^{-2}	leucine-rich transmembrane protein
AAEL003859-RA	-0.0278	1.0×10^{-0}	leucine-rich transmembrane protein
AAEL004466-RA	0.4468	1.0×10^{-0}	leucine-rich immune protein (Coil-less)
AAEL004773-RA	-0.0228	1.0×10^{-0}	leucine carboxyl methyltransferase
AAEL005351-RA	0.1178	6.8×10^{-1}	leucine-rich transmembrane protein
AAEL005734-RA	0.2204	5.8×10^{-1}	leucine-rich transmembrane protein
AAEL005762-RA	0.5146	7.0×10^{-1}	leucine-rich transmembrane protein
AAEL006026-RA	-0.0849	9.3×10^{-1}	leucine rich protein, putative
AAEL006377-RA	-0.8786	8.4×10^{-1}	leucine-rich immune protein (Coil-less)
AAEL006975-RA	2.0656	1.0×10^{-2}	leucine aminopeptidase
AAEL007103-RA	-0.5660	9.0×10^{-1}	leucine-rich immune protein TM
AAEL007224-RA	0.0491	1.0×10^{-0}	leucine-rich immune protein (Coil-less)
AAEL007231-RA	-2.5365	4.8×10^{-1}	leucine-rich immune protein (Coil-less)
AAEL007363-RA	0.3441	6.6×10^{-1}	leucine-rich transmembrane protein
AAEL007565-RA	0.0044	1.0×10^{-0}	leucine rich protein, putative
AAEL007778-RA	-1.4858	3.1×10^{-1}	leucine-rich transmembrane protein
AAEL007785-RA	0.5315	8.3×10^{-1}	leucine-rich transmembrane protein
AAEL008658-RA	0.3609	8.9×10^{-1}	leucine-rich immune protein TM
AAEL009520-RA[1]	-0.9495	7.8×10^{-1}	leucine-rich immune protein (Long)
AAEL009792-RA	-1.2373	6.2×10^{-1}	leucine-rich immune protein (Coil-less)

Transcript ID	Log2FC	p-adj	Gene description
AAEL009894-RA	0.8665	9.7×10^{-5}	leucine-rich immune protein (Coil-less)
AAEL010111-RA	-0.2184	9.6×10^{-1}	leucine-rich transmembrane protein
AAEL010125-RA	-1.5972	4.5×10^{-1}	leucine-rich immune protein (Coil-less)
AAEL010128-RA	-0.0478	1.0×10^{-0}	leucine-rich immune protein (Long)
AAEL010132-RA	-1.7077	3.7×10^{-1}	leucine-rich immune protein (Long)
AAEL010286-RA	-3.3918	5.0×10^{-9}	leucine-rich transmembrane protein
AAEL010656-RA	0.2515	9.6×10^{-1}	leucine-rich immune protein (Short)
AAEL010772-RA	-0.3624	9.7×10^{-1}	Leucine-rich repeat-containing protein 50 homolog
AAEL011387-RA	-0.1587	9.4×10^{-1}	leucine-rich repeat protein
AAEL011760-RA	-0.9940	4.4×10^{-1}	leucine-rich transmembrane protein
AAEL012086-RA	-1.4555	6.0×10^{-1}	leucine-rich immune protein (Long)
AAEL012092-RA	0.5027	4.8×10^{-1}	leucine rich repeat protein
AAEL012093-RA	0.9895	7.1×10^{-2}	leucine-rich transmembrane protein
AAEL012255-RA	-0.4482	9.4×10^{-1}	leucine-rich immune protein (Short)
AAEL012538-RA	-0.1787	1.0×10^{-1}	leucine-rich immune protein (Short)
AAEL012763-RA	-1.1587	1.5×10^{-1}	leucine-rich immune protein (Coil-less)
AAEL012767-RA	-2.6500	8.8×10^{-2}	leucine-rich immune protein (Short)
AAEL012771-RA	-1.2948	6.3×10^{-1}	leucine-rich immune protein (Coil-less)
AAEL012911-RA	0.8990	2.6×10^{-1}	leucine-rich immune protein (Coil-less)
AAEL015627-RA	-0.1380	1.0×10^{-0}	leucine-rich immune protein (Coil-less)

[1] AAEL009520-RA is the same gene as AAEL024406.

Table S1D. Female *Aedes aegypti* transcriptomic RNA-seq data show Leucine-Rich Repeat Proteins related genes dysregulated in the Orlando strain *Aedes aegypti* 7-days post infection with ZIKV compared with Control (uninfected blood-feeding only) in Orlando strain.

Transcript ID	Log2FC	p-adj	Gene description
AAEL000108-RA	-2.3592	3.5×10^{-1}	leucine aminopeptidase
AAEL000243-RA	0.7358	7.8×10^{-1}	leucine-rich transmembrane protein
AAEL000424-RA	0.0654	8.7×10^{-1}	leucine aminopeptidase
AAEL000925-RA	0.0531	9.7×10^{-1}	leucine-zipper-like transcriptional regulator
AAEL001401-RA	-3.3173	6.3×10^{-2}	leucine-rich immune protein (Short)
AAEL001402-RA	-3.3550	6.1×10^{-5}	leucine-rich immune protein (Short)
AAEL001414-RA	-3.6456	5.0×10^{-2}	leucine-rich immune protein (Short)
AAEL001417-RA	-4.3471	7.7×10^{-2}	leucine-rich immune protein (Short)
AAEL001420-RA	-3.9058	4.5×10^{-2}	leucine-rich immune protein (Short)
AAEL001649-RA	0.1785	5.5×10^{-1}	leucine aminopeptidase
AAEL001766-RA	-1.7846	1.9×10^{-1}	leucine-rich transmembrane protein
AAEL002166-RA	0.0738	9.0×10^{-1}	leucine rich repeat (in flii) interacting protein
AAEL002295-RA	-2.2315	3.4×10^{-2}	leucine-rich transmembrane protein
AAEL002307-RA	-1.6840	4.1×10^{-2}	leucine-rich transmembrane protein
AAEL002615-RA	-1.9453	3.0×10^{-1}	leucine-rich transmembrane protein
AAEL003262-RA	-0.3746	9.6×10^{-1}	leucine-rich transmembrane protein
AAEL003408-RA	-0.5226	7.4×10^{-1}	leucine-rich transmembrane protein
AAEL003554-RA	0.0152	1.0×10^{-0}	leucine rich repeat protein

Transcript ID	Log2FC	p-adj	Gene description
AAEL003597-RA	-1.1217	9.3×10^{-1}	leucine-rich transmembrane protein
AAEL003597-RB	-1.4416	6.3×10^{-1}	leucine-rich transmembrane protein
AAEL003713-RA	-1.4416	6.3×10^{-1}	leucine-rich transmembrane protein
AAEL003720-RA	-1.2985	4.1×10^{-1}	leucine-rich transmembrane protein
AAEL003859-RA	-1.1459	2.6×10^{-1}	leucine-rich transmembrane protein
AAEL004466-RA	0.0440	1.0×10^{-0}	leucine-rich immune protein (Coil-less)
AAEL004773-RA	-0.1240	1.0×10^{-0}	leucine carboxyl methyltransferase
AAEL005351-RA	0.0501	9.8×10^{-1}	leucine-rich transmembrane protein
AAEL005734-RA	0.0344	9.8×10^{-1}	leucine-rich transmembrane protein
AAEL005762-RA	-0.0691	9.5×10^{-1}	leucine-rich transmembrane protein
AAEL006026-RA	-2.1727	1.2×10^{-1}	leucine rich protein, putative
AAEL006377-RA	0.0329	1.0×10^{-0}	leucine-rich immune protein (Coil-less)
AAEL006975-RA	-2.1250	1.9×10^{-2}	leucine aminopeptidase
AAEL007103-RA	-1.1907	2.6×10^{-1}	leucine-rich immune protein TM
AAEL007224-RA	-2.3203	1.9×10^{-2}	leucine-rich immune protein (Coil-less)
AAEL007231-RA	0.5661	9.6×10^{-1}	leucine-rich immune protein (Coil-less)
AAEL007363-RA	-3.0437	1.6×10^{-1}	leucine-rich transmembrane protein
AAEL007565-RA	-0.8595	3.8×10^{-2}	leucine rich protein, putative
AAEL007778-RA	-0.0497	9.6×10^{-1}	leucine-rich transmembrane protein
AAEL007785-RA	-1.8464	1.3×10^{-2}	leucine-rich transmembrane protein
AAEL008658-RA	-0.9593	5.4×10^{-1}	leucine-rich immune protein TM
AAEL009520-RA[1]	-1.7802	8.1×10^{-2}	leucine-rich immune protein (Long)
AAEL009792-RA	-2.2074	2.8×10^{-4}	leucine-rich immune protein (Coil-less)
AAEL009894-RA	0.0551	9.9×10^{-1}	leucine-rich immune protein (Coil-less)
AAEL010111-RA	-0.3721	9.2×10^{-1}	leucine-rich transmembrane protein
AAEL010125-RA	-3.2166	2.2×10^{-2}	leucine-rich immune protein (Coil-less)
AAEL010128-RA	-5.3692	2.8×10^{-6}	leucine-rich immune protein (Long)
AAEL010132-RA	-0.7796	8.7×10^{-1}	leucine-rich immune protein (Long)
AAEL010286-RA	-1.9108	5.4×10^{-1}	leucine-rich transmembrane protein
AAEL010656-RA	-2.5623	4.4×10^{-3}	leucine-rich immune protein (Short)
AAEL010772-RA	-0.8164	9.2×10^{-1}	Leucine-rich repeat-containing protein 50 homolog
AAEL011387-RA	-0.2728	8.2×10^{-1}	leucine-rich repeat protein
AAEL011760-RA	1.2954	3.9×10^{-1}	leucine-rich transmembrane protein
AAEL012086-RA	-1.7527	2.6×10^{-2}	leucine-rich immune protein (Long)
AAEL012092-RA	-1.9051	1.2×10^{-1}	leucine rich repeat protein
AAEL012093-RA	-1.8185	2.4×10^{-2}	leucine-rich transmembrane protein
AAEL012255-RA	-4.1624	3.1×10^{-5}	leucine-rich immune protein (Short)
AAEL012763-RA	-1.1534	1.8×10^{-1}	leucine-rich immune protein (Coil-less)
AAEL012767-RA	-0.6035	9.8×10^{-1}	leucine-rich immune protein (Short)
AAEL012771-RA	3.4269	5.6×10^{-2}	leucine-rich immune protein (Coil-less)
AAEL012911-RA	-0.2531	9.4×10^{-1}	leucine-rich immune protein (Coil-less)

[1] AAEL009520-RA is the same gene as AAEL024406.

Figure S1. Evolutionary analysis of paralogues of *AeaAPL1* of the *Ae. Aegypti*.

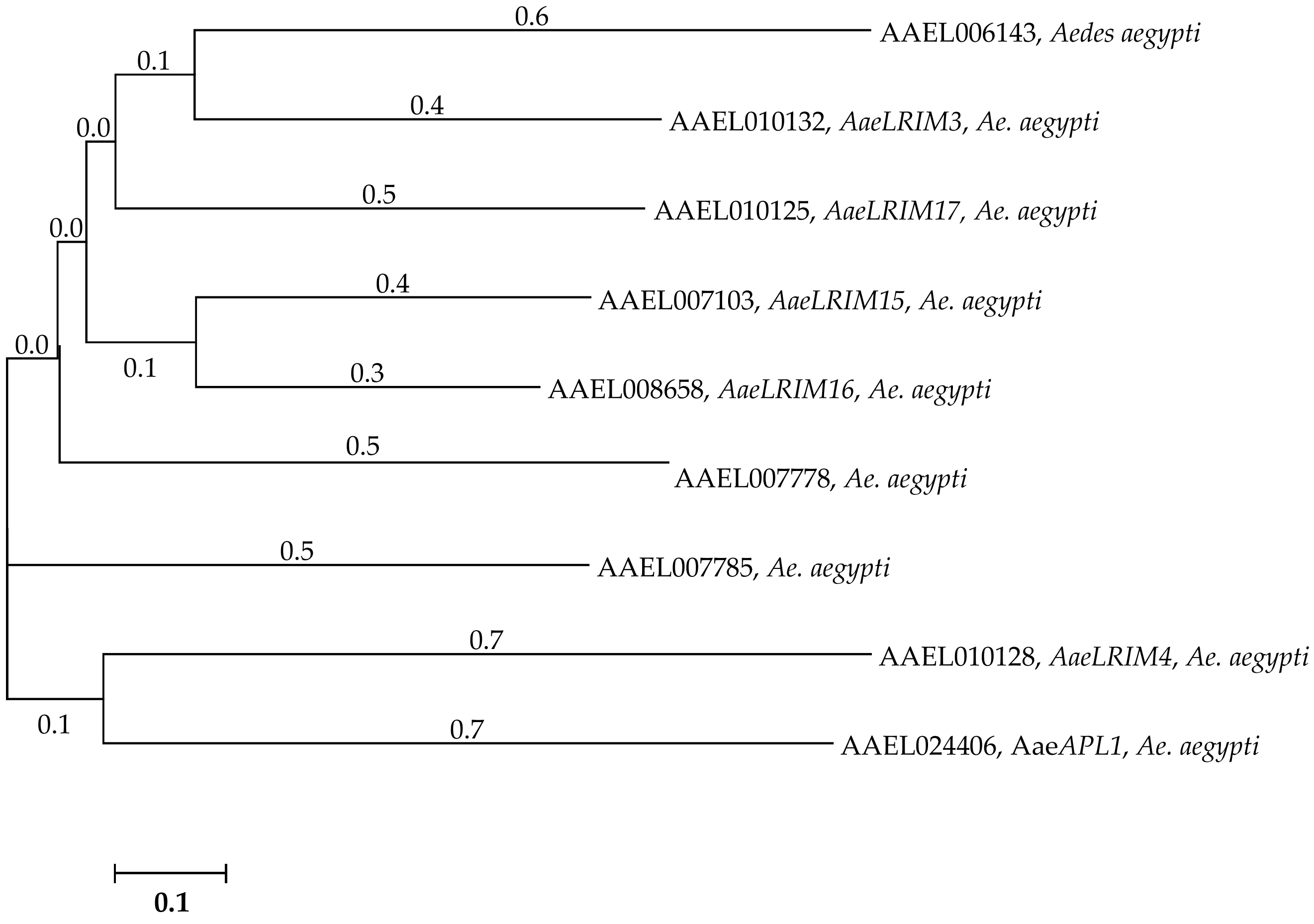

Figure S1. The evolutionary history was concluded by means of the Neighbor-Joining method [1]. The optimal tree with the sum of branch length = 4.948 is revealed. The tree is drawn to scale, with branch lengths in the same units as those of the evolutionary distances used to infer the phylogenetic tree. The evolutionary distances were calculated with the Maximum Composite Likelihood method [2] and are in the units of the number of base substitutions per site. The analysis contained 9 nucleotide sequences. All positions containing gaps and missing data were disregarded. The final dataset had a total of 1179 positions. Evolutionary analyses were performed in MEGA7 [3].

Saitou N. and Nei M. (1987). The neighbor-joining method: A new method for reconstructing phylogenetic trees. Molecular Biology and Evolution 4:406-425.

2. Tamura K., Nei M., and Kumar S. (2004). Prospects for inferring very large phylogenies by using the neighbor-joining method. Proceedings of the National Academy of Sciences (USA) 101:11030-11035.

3. Kumar S., Stecher G., and Tamura K. (2016). MEGA7: Molecular Evolutionary Genetics Analysis version 7.0 for bigger datasets. Molecular Biology and Evolution 33:1870-1874.

Figure S2A-E. *AaeLRIM3*, *AaeLRIM4*, *AaeLRIM15*, *AaeLRIM16*, and *AaeLRIM17* relative expression in the all developmental stages.

A

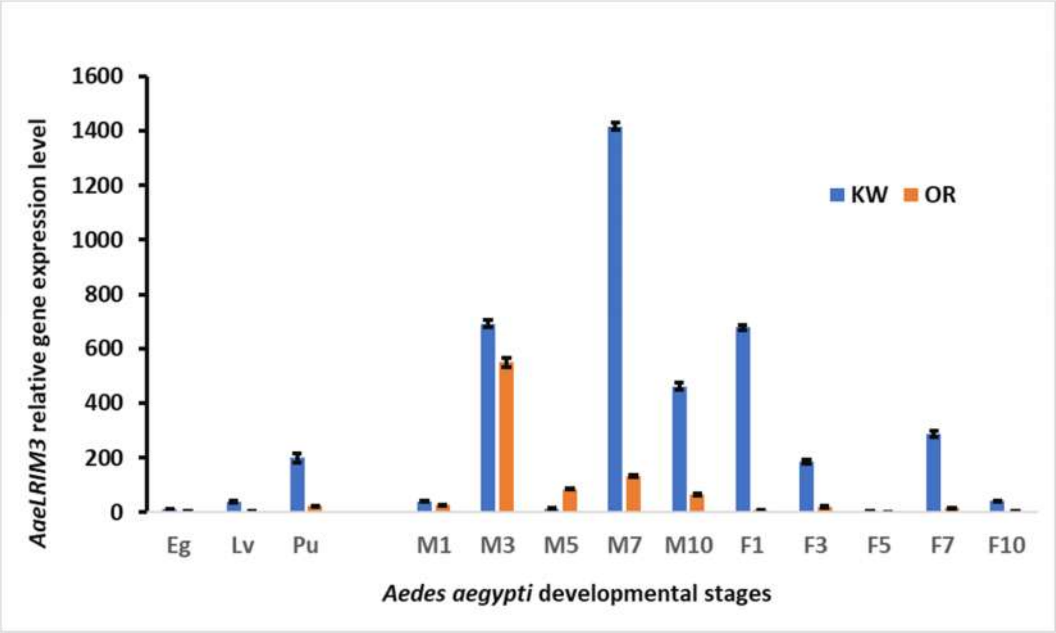

Figure S2A. *AaeLRIM3* relative expression in the all developmental stages.

B

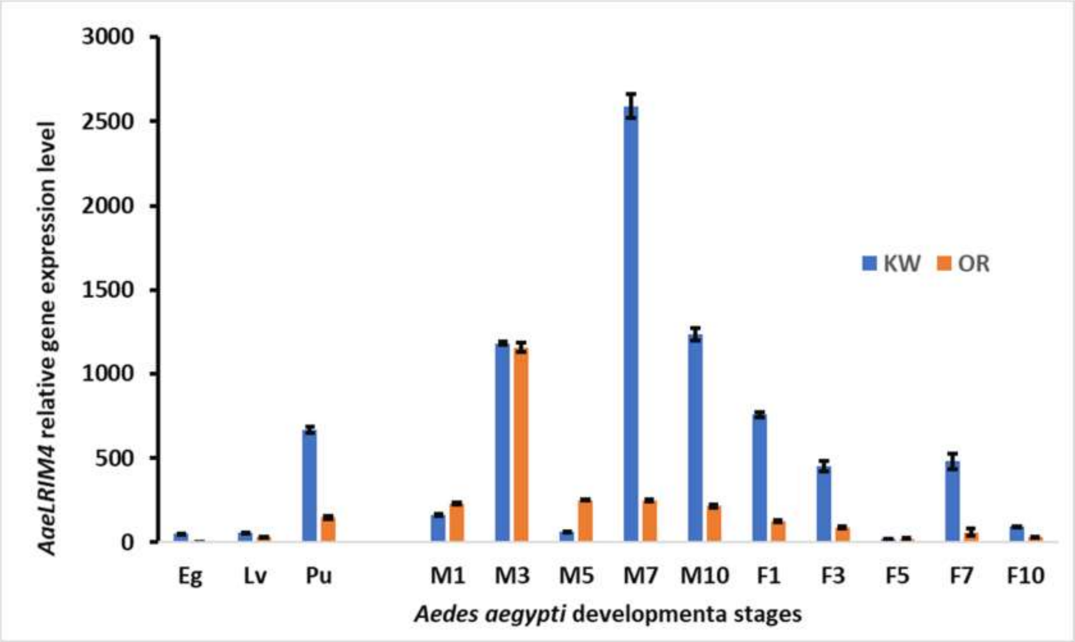

Figure S2B. *AaeLRIM4* relative expression in the all developmental stages.

C

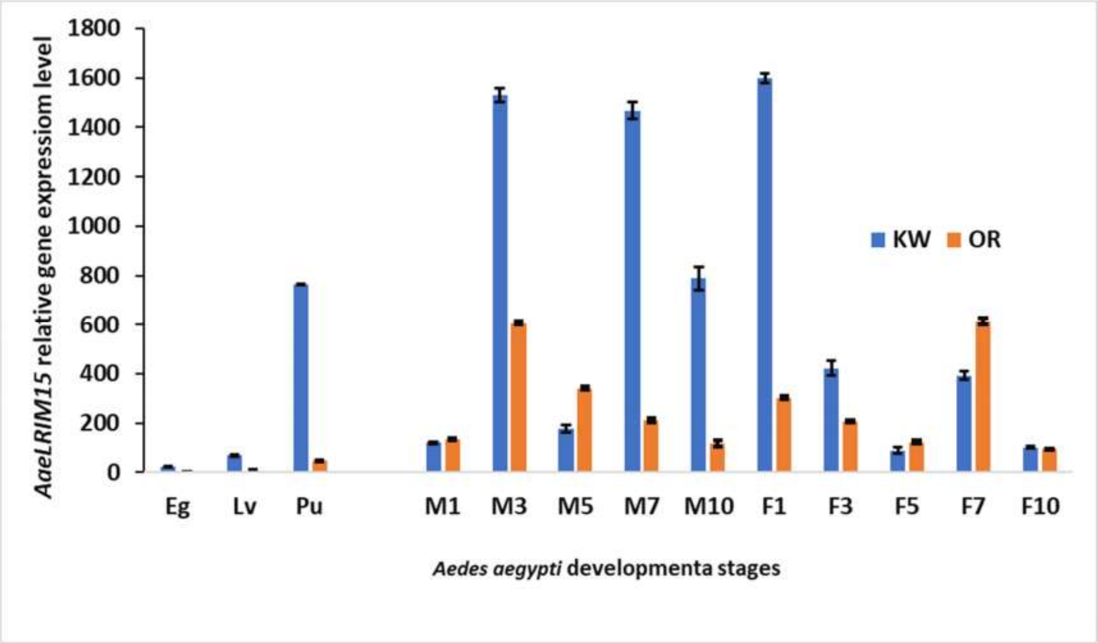

Figure S2C. *AaeLRIM15* relative expression in the all developmental stages.

D

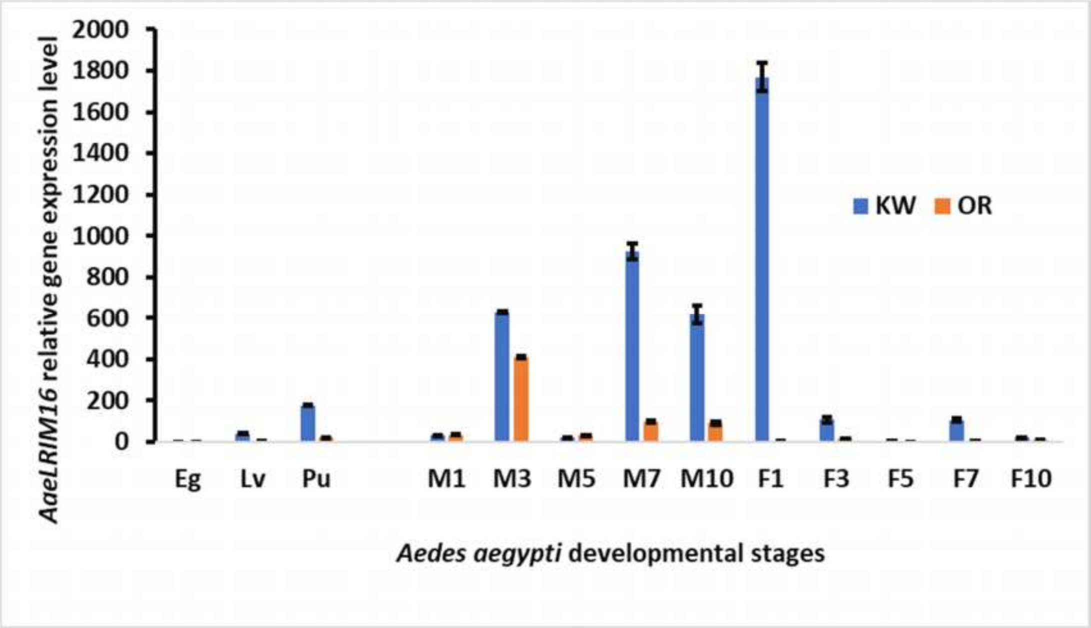

Figure S2D. *AaeLRIM16* relative expression in the all developmental stages.

E

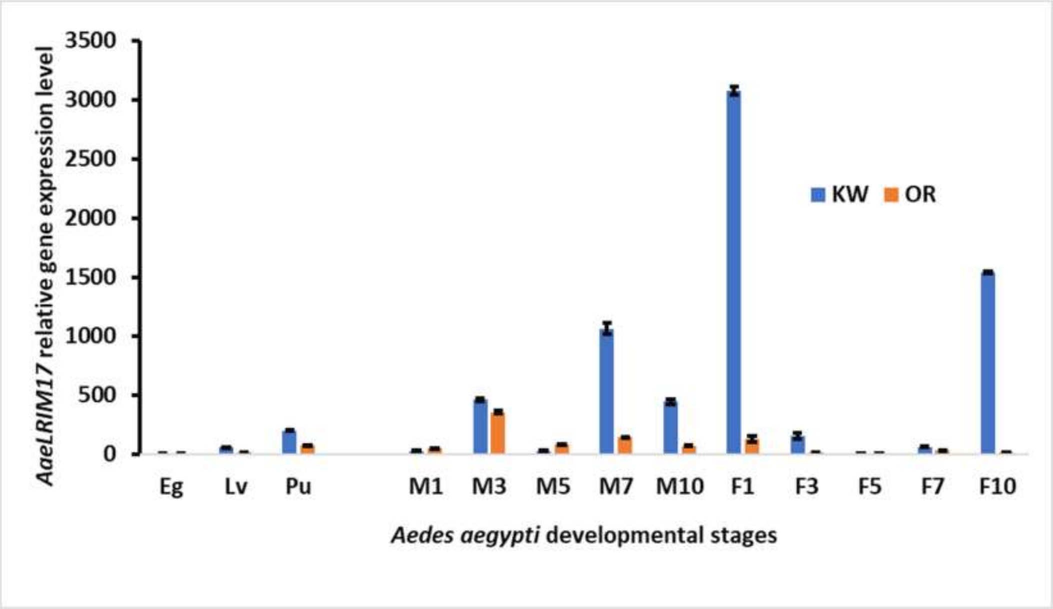

Figure S2E. *AaeLRIM17* relative expression in the all developmental stages.

Figure S2A-E. *AaeLRIM3, AaeLRIM4, AaeLRIM15, AaeLRIM16,* and *AaeLRIM17* relative expression in the all developmental stages, from eggs (Eg), larvae (Lv), pupae (Pu), and adults *Ae. aegypti*, including male (M1, male 1-d-old; M3, male 3-d-old; M5, male 5-d-old; M7, male 7-d-old; and M10, male 10-d-old) and female (F1, female 1-d-old; F3, female 3-d-old; F5, female 5-d-old; F7, female 7-d-old; and 10F, female 10-dold) in the Key West strain (KW) and Orlando (OR) strain *Aedes aegypti*. (**A**) *AaeLRIM3*; (**B**) *AaeLRIM4;* (**C**) *AaeLRIM15;* (**D**) *AaeLRIM16;* and (**E**) *AaeLRIM17.*
